# Evolution of an Early and Long-Lasting Iron Smelting Technique at Didé West 1, Falémé Valley, Eastern Senegal

**DOI:** 10.1007/s10437-026-09653-z

**Published:** 2026-03-25

**Authors:** Mélissa Morel, Pierre Lamotte, Ladji Dianifaba, Alexander Walmsley, Irka Hajdas, Vincent Serneels, Anne Mayor

**Affiliations:** 1 Faculty of Sciences, Section of Biology, Laboratory ARCAN, University of Geneva, Geneva, Switzerland; 2 Faculty of Letters and Human Sciences, Department of History, University Cheikh Anta Diop, Dakar, Senegal; 3 Film University Konrad Wolf, Potsdam, Germany; 4 Department of Earth and Planetary Sciences, Laboratory Ion Beam Physics, ETHZ, Zurich, Switzerland; 5 Faculty of Sciences and Medecine, Department of Earth Sciences, Institute of Geology, University of Fribourg, Fribourg, Switzerland; 6 University of Geneva, Global Studies Institute, Geneva, Switzerland; 7 McDonald Institute for Archaeological Research, University of Cambridge, Cambridge, United Kingdom

**Keywords:** Iron metallurgy, Eastern Senegal, Early Iron Age, Technical choices, Archaeology, Métallurgie du fer, Sénégal oriental, Premier âge du Fer, Choix techniques, Archéologie

## Abstract

The region west of the Falémé Valley in eastern Senegal, where several distinct smelting traditions have been identified, is key to understanding early West African iron metallurgy, which remains globally underdocumented in this part of the continent. Excavations carried out in 2018 and 2022 at the Didé West 1 site (DDW1) uncovered an exceptionally well-preserved slag heap dated between the 4th century BCE and the 4th century CE, representing nearly eight centuries of activity. The site belongs to the FAL02 smelting tradition and revealed 35 circular clay-lined furnace bases producing internal slag blocks, numerous massive multiperforated tuyeres, and the distinctive use of palm nuts as stuffing material. This outstanding preservation offers a unique opportunity to investigate long-term technological continuity and adaptation. The stratigraphic and spatial analyses made it possible to reconstruct both the chronological sequence and the evolution of the workshop’s layout, which gradually shifted northward over time. Quantitative estimations suggest that iron smelting was likely small-scale and seasonal, primarily aimed at meeting local needs. Despite its extraordinary longevity, the tradition remained remarkably stable, with only minor technical adjustments. This technique and its continuity contrast with other metallurgical contexts and highlight the significance of technological choices in early iron production.

## Résumé

La région située à l’ouest de la vallée de la Falémé, au Sénégal oriental, où plusieurs traditions de réduction du fer ont été identifiées, constitue une zone clé pour la compréhension des origines de la métallurgie du fer en Afrique de l’Ouest, encore largement sous-documentée dans cette partie du continent. Les fouilles menées en 2018 et 2022 sur le site de Didé West 1 (DDW1) ont mis au jour un amas de scories exceptionnellement bien conservé, daté entre le IVᵉ siècle avant notre ère et le IVᵉ siècle de notre ère, correspondant à près de huit siècles d’activité métallurgique. Le site appartient à la tradition technique FAL02 et a révélé 35 bases de fourneaux circulaires associées à des blocs de scories internes, de nombreuses tuyères massives à perforations transversales, ainsi que l’usage inhabituel suppress de noix de palmier rônier comme matériau de bourrage. Cet état de conservation remarquable offre une occasion unique d’étudier la continuité et l’adaptation d’une technique sidérurgique sur le long terme. Les analyses stratigraphiques et spatiales ont permis de reconstituer la séquence chronologique et l’évolution de l’organisation de l’atelier, qui se déplace progressivement vers le nord au fil du temps. Les estimations quantitatives suggèrent une production à petite échelle et saisonnière, principalement destinée à répondre aux besoins locaux. Malgré son exceptionnelle longévité, cette tradition technique est restée remarquablement stable, ne présentant que des ajustements mineurs. Cette continuité contraste avec d’autres contextes métallurgiques et souligne l’importance des choix techniques dans les débuts de la sidérurgies ouest africaine.

## Introduction

In West Africa, a growing number of iron production sites attest to the use of diverse smelting techniques as early as the first millennium BCE (Robion-Brunner, 2018). However, only a few of these sites are sufficiently well preserved, well documented, and well dated to allow for in-depth analysis of the historical dynamics and anthropological dimensions of this early period of iron production. It is even rarer to identify early smelting sites that show long-term use and a well-established chronological sequence. Such sites are essential for investigating how technical practices evolved over time, how workshops were spatially organized, and how smelters adapted their choices in response to changing needs or constraints in raw materials.

The origins of iron metallurgy in West Africa have been the subject of extensive debate and literature review (e.g., Alpern, 2005; Holl, 2009; Killick, 2004; Robion-Brunner, 2018), which we will not revisit here. Current research indicates the existence of several early iron production districts in sub-Saharan West Africa, dating to the first millennium BCE. These districts are often geographically isolated from one another, and their potential interconnections remain poorly understood. The number of sites with firmly established chronologies that are broadly accepted by the scholars’ community remains relatively limited.

From east to west, the Nsukka region in Nigeria has long been considered a major center for early ironworking in sub-Saharan Africa. However, direct evidence for sustained iron production there remains limited (Okafor, 2002; Okafor & Phillips, 1992). Sites on the Jos Plateau, dated to around 600 BCE, further demonstrate the early emergence of iron metallurgy in this region (Junius, 2023). In Niger, the Termit Massif has yielded evidence of iron production dating to the early first millennium BCE, though claims of significantly earlier metallurgy remain controversial (Killick et al., 1988; Quéchon & Roset, 1974). Further west, early iron smelting is attested in Burkina Faso around 700 BCE at Douroula (Holl, 2019; Kiethega, 2006) and in Togo by 400 BCE, as shown by recent excavations at Dekpassanware (de Barros et al., 2020).

Senegal is among the few West African countries with confirmed evidence of early primary iron metallurgy. The Senegal River Valley is one of its best already documented metallurgical districts. The earliest evidence of iron production located in the middle Senegal River Valley comes from the site of Walaldé, where isolated slag and tuyère fragments have been dated between 800 and 550 BCE (Deme, 2003; Deme & McIntosh, 2006). A total of 19 kg of slag and 47 tuyères were recovered, confirming metallurgical activity, though on a modest scale. No furnaces have been identified, making technological reconstructions speculative at best. Based on slag morphology, Killick suggested the use of small furnaces with internal diameters of 40 cm or less, capable of reaching temperatures high enough to produce steel (Killick in Deme, 2003; Killick, 2016). Nonetheless, the lack of well-preserved smelting structures leaves many aspects of the process unresolved.

Several iron production sites located on the Senegalese bank of the river, including Juudé Jaabé, Sincaan, also feature alignments of slag-pit furnaces. But radiocarbon dates place their activity in the first half of the second millennium CE (Deme & McIntosh, 2006). Moreover, the sheer number of furnaces should not be taken as evidence of large-scale production, as all these structures appear to have been single-use, producing only a few kilograms of metal per smelt. A systematic survey conducted in 1983 on the Mauritanian side of the Senegal River recorded over 40,000 single-use furnace bases along an 80 km stretch (Robert-Chaleix & Sogane 1983; MacDonald et al. 2009). This abundance is likely due to ore sources being concentrated on the Mauritanian bank of the river (Killick, 2016). Unfortunately, these excavations suffer from poor dating, leaving us with an incomplete understanding of these sites. As far as we know, metallurgical remains do not correspond to early ironworking but are instead associated with iron production from the early second millennium CE.

Finally, apart from scant evidence at Walaldé, most of the documented sites from the Senegal River Valley date to the second millennium CE and remain poorly understood (Dianifaba, 2020; Thiaw, 1999).

A persistent challenge in the study of early metallurgy in West Africa lies in the poor preservation of remains, which are often scattered, fragmentary, and difficult to interpret. This greatly limits our ability to reconstruct ancient techniques and evaluate patterns of technological continuity across regions. One of the most significant obstacles is obtaining reliable radiocarbon dates. Charcoal is often scarce at these sites, and when it is present, early phases frequently fall within the plateau of the calibration curve during the first millennium BCE, reducing chronological precision (Killick, 2004; Robion-Brunner, 2018). Additionally, unexplained chronological gaps between the earliest radiocarbon dates and subsequent production phases are common. As archaeometallurgists have consistently emphasized, refining our understanding of early African iron metallurgy will depend on extensive, high-resolution fieldwork supported by rigorous dating protocols and well-contextualized material analyses.

In eastern Senegal, new evidence for early iron production has been discovered in the Falémé River Valley and its western neighborhood, further upstream from the Senegal River. Since 2012, research conducted by Anne Mayor and her team within the framework of the project *Human Population and Paleoenvironment in Africa – Falémé* has significantly advanced our knowledge of this region’s metallurgical past. Intensive surveys along the valley have identified numerous iron production sites, with notable concentrations around Saraya in the south and Koussan in the north (Huysecom et al., 2013, 2014, 2015, 2016). Targeted excavations carried out in 2017 and 2018 west of the Falémé Valley, in the RNCB (Réserve naturelle Communautaire du Boundou), revealed four distinct iron smelting traditions, dated between 400 BCE and 700 CE (Walmsley, 2018; Walmsley et al., 2020). Although still under study, these traditions show considerable technical variability despite their geographic proximity, raising important questions about their chronological and cultural relationships.

**Fig. 1:**
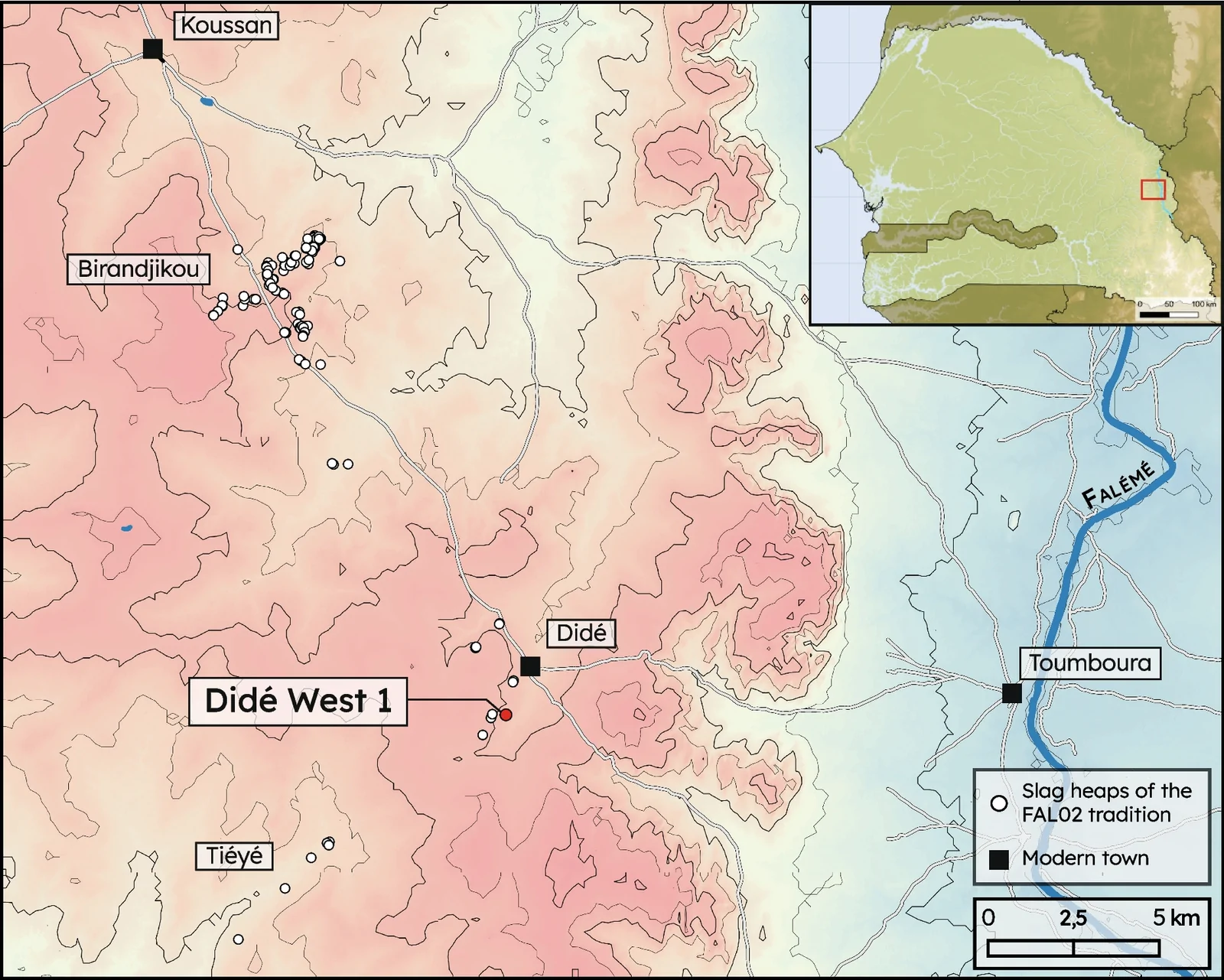
Map of the study area southeast of Koussan. The slag heaps associated with the FAL02 technical tradition are marked. The Didé West 1 slag heap, which is the primary focus of this article, is highlighted in red (P. Lamotte)

Among these, the smelting tradition designated as FAL02 (referred to as Group 2 in Walmsley et al., 2020), located between the villages of Koussan and Didé, stands out due to both its early chronology—extending into the second half of the first millennium BCE—and the excellent preservation of its remains. Unlike sites such as Walaldé, where only scattered slag fragments were recovered (Deme & McIntosh, 2006), the FAL02 tradition is characterized by numerous slag heaps (Fig. 1) and the presence of in situ furnace bases. These features offer a rare opportunity to investigate early iron production in detail.

The Didé West 1 (DDW1) site represents the most well-preserved and most ancient example of the FAL02 tradition. Excavations of this large slag heap revealed an alignment of 35 furnace bases, spanning approximately 800 years of metallurgical activity. This site is remarkable not only for its early chronology and exceptional preservation but also for the extensiveness of the excavation. These conditions make DDW1 a key reference for reconstructing technological practices and assessing how smelting techniques evolved—or remained stable—over a long period of time.

This paper presents a comprehensive analysis of the DDW1 site, focusing on the reconstruction of the smelting process and the degree of standardization within the FAL02 tradition. Through a detailed examination of the site’s stratigraphy, spatial organization, and metallurgical remains, this study contributes to broader discussions on the variability and organization of early iron production in western Africa.

## Methods

### Research Context and Excavation Campaigns

Following the initial fieldwork conducted in 2017 west of the Falémé Valley, further investigations were necessary to better understand the early smelting traditions identified in the region. In 2018, a targeted survey was carried out in collaboration with the eco-guard and blacksmith Abdoulaye Kanté from Koussan. This led to the discovery of the slag heap of Didé West 1 (DDW1), located approximately 3 km west of the current village of Didé. DDW1 stood out immediately due to its large size and the fact that it had not been disturbed by farming.

Initial excavations were conducted in 2018 immediately after the discovery of the heap. The primary aim was to study the slag assemblage and to compare it with the iron smelting traditions identified the year before. A trench was excavated from the periphery toward the center of the slag heap, eventually revealing an unusual double semicircular alignment of dozens of suppress well-preserved massive tuyeres (Figs. 3 and 7). Although no furnaces were identified during this campaign, the first radiocarbon and thermoluminescence dates confirmed an early and coherent chronology during the first millennium BCE (Mayor et al., 2021).

Between 2020 and 2021, systematic surveys were conducted by Pierre Lamotte between Koussan and Didé to better understand the spatial distribution of FAL02 smelting sites. These surveys, complemented by surface observations, led to the identification of over 130 slag heaps associated with this technical tradition (Fig. 1). These sites are generally located 15–30 km away from the Falémé River, a pattern that suggests a deliberate choice to smelt iron near ore sources rather than close to water. Three additional slag heaps were excavated in the Birandjikou sector (Fig. 1) to clarify the spatial, chronological, and technical aspects of the FAL02 tradition. These excavations led to the first discovery of furnaces associated with this tradition, located on the periphery of the slag heaps (Mayor et al., 2021, 2022).

In 2022, a new excavation was conducted at DDW1 to uncover furnace structures and refine the site’s chronology. Several small test pits were dug around the slag heap until furnaces were located on its northern edge. A trench was then opened from that point toward the center of the slag heap, connecting to the 2018 trench. In total, 35 furnace bases were discovered in situ, and a total of 47 m^2^ were excavated across the two campaigns, distributed among eight test pits and trenches (Fig. 3). Photogrammetry was systematically used to document excavation contexts, including all trenches and furnace bases (Mayor et al., 2023).

Compared to the other surveyed slag heaps in the sector of Birandjikou, which yielded fewer well-preserved remains, DDW1 offers a rare and comprehensive perspective on this technical tradition, making it a key site for understanding early iron metallurgy in eastern Senegal.

### Ore Collection and Mining Evidence

**Fig. 2:**
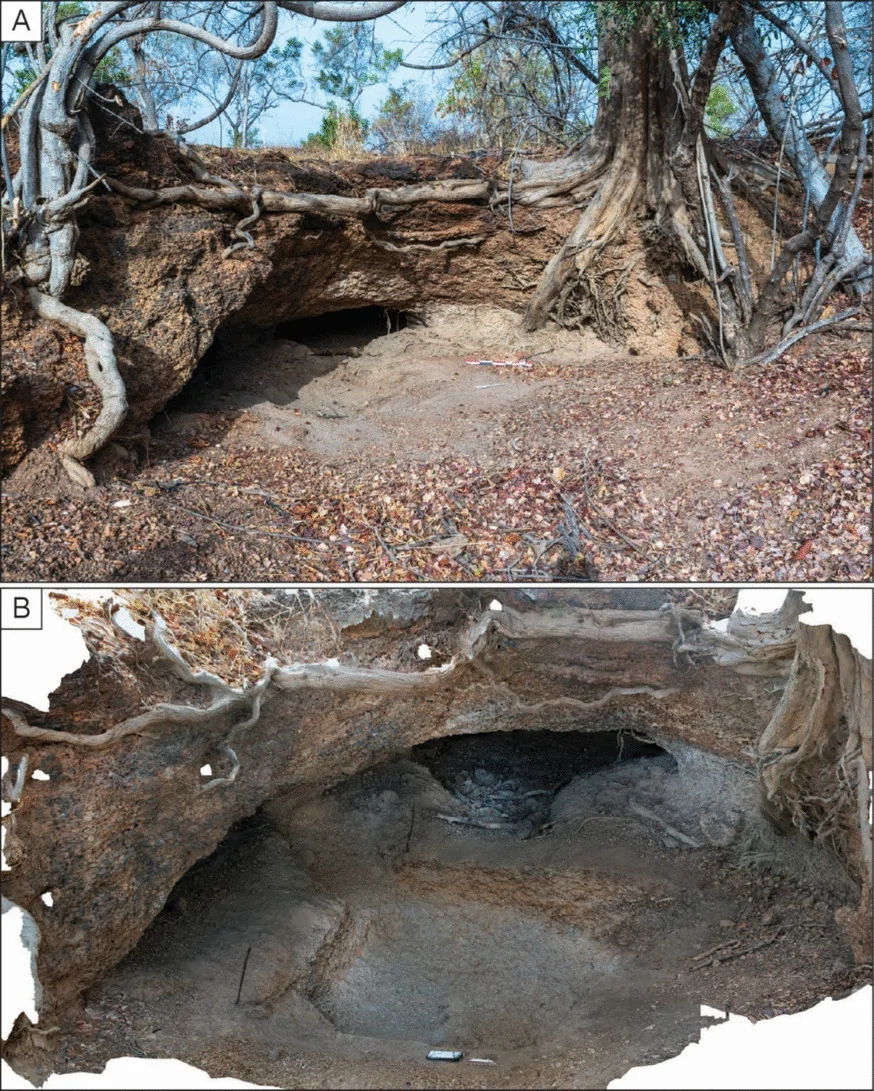
Photo of the iron mine DID_015 before excavation (**A**). Photogrammetry of the test pit DID_015 (**B**) (P. Lamotte, from Mayor et al., 2023)

In 2022, surveys have been conducted to locate ore extraction areas. A few hundred meters east of the DDW1 slag heap, erosion has exposed openings dug beneath the lateritic crust, which appear to have been intentionally excavated (Mayor et al., 2023). This aligns with reports of laterite exploitation in other parts of the Senegal River region (Bocoum 2002; Killick, 2016). Excavations at one of these features (DID_015, Fig. 2) have uncovered a significant accumulation of potential iron ore, sterile rock, and stone tools used for percussion and grinding. Preliminary analyses of tool marks, heating traces on the walls, and associated archaeological material suggest that this could be a mining area, though further confirmation is needed. Ore has been collected and is currently being analyzed at the Department of Geosciences of the University of Fribourg (Morel et al., in preparation).

### Recording and Documentation Methods

On-site, nonfragmented slag blocks were photographed, measured, and weighed. They were classified into distinct morphological groups. Tuyeres were systematically documented through photogrammetry, and measurements were taken from these 3D models to estimate average dimensions and variability. Excavation trenches and furnace bases were also recorded using photogrammetry. Dimensions (length, width, and depth) of the furnace bases were extracted from the 3D models for detailed analysis.

### Quantifying Production and Laboratory Analysis

To estimate production intensity, the volume of the slag heap was evaluated during fieldwork. Three one-cubic-meter sections of the heap were excavated, sieved, sorted, and weighed to determine the average mass of slag per cubic meter. This approach allowed for the estimation of the total slag mass, following the methodology proposed by Robion-Brunner and Serneels (2017). These data provide a basis for approximating the number of smelting events and, more broadly, for reflecting on the organization and intensity of iron production at the site.

In parallel, following the methodology developed at the University of Fribourg, more than 100 slag and ore samples from DDW1 and other sites associated with the FAL02 tradition are currently undergoing global chemical analysis using WD-XRF. The results of these analyses will be presented in a separate publication (Morel et al., in preparation).

## Results

### The Chrononology of Didé West 1

Didé West 1 (DDW1) is not only one of the largest slag heaps associated with the FAL02 technical tradition but also one of the earliest dated metallurgical workshops in the region—alongside the FAL01 tradition documented at Madina Cheikh Oumar 1 (Mayor et al., 2025; Walmsley et al., 2020). Radiocarbon and OSL analyses reveal a continuous yet evolving smelting tradition on this workshop, spanning at least 800 years, from the 4th century BCE to the 4th century CE (Mayor et al., 2022 – Fig. 4). This longevity is unprecedented in the area and challenges existing narratives.

**Fig. 3:**
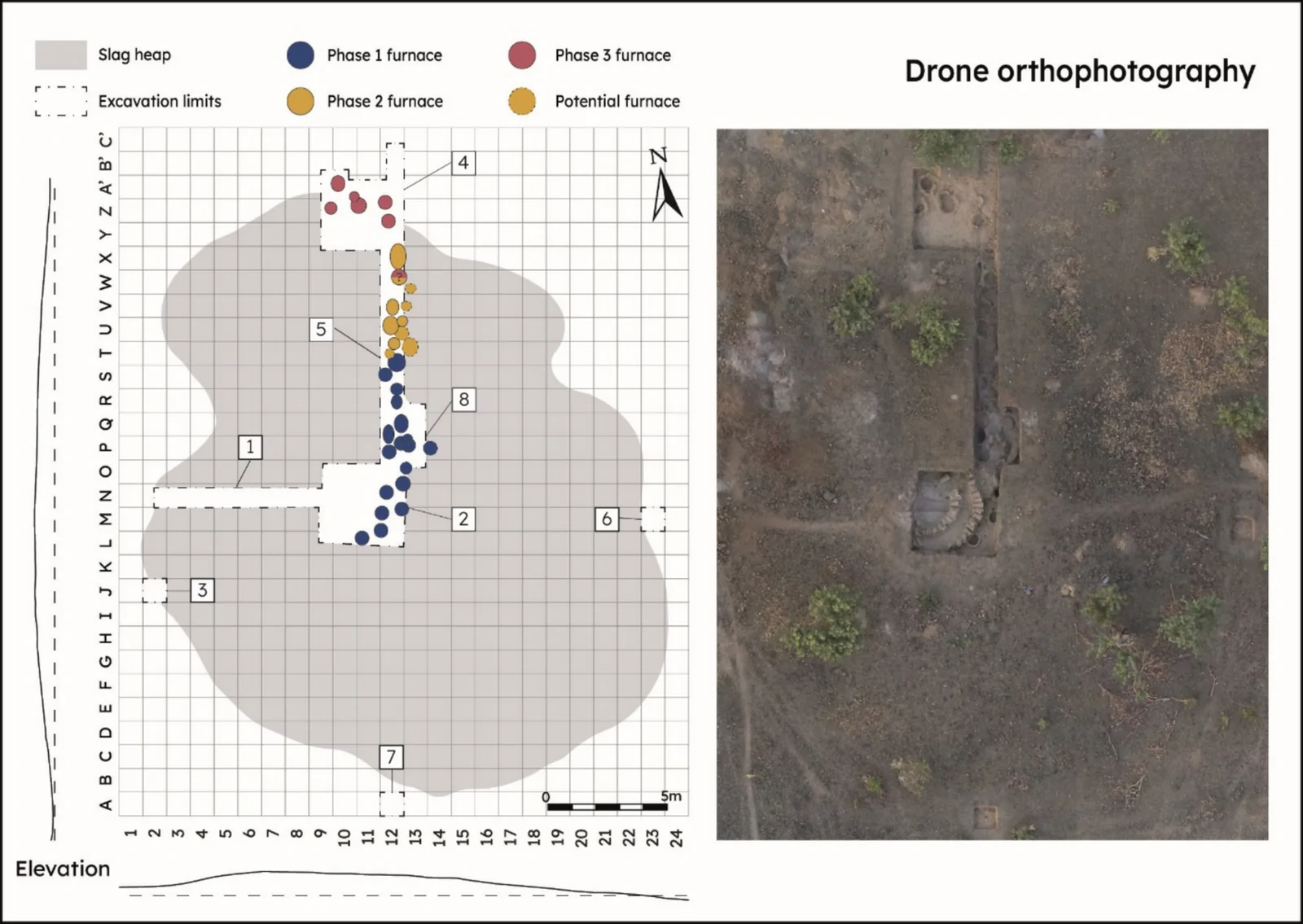
Plan of the DDW1 slag heap and its elevation. The numbers on the plan indicate the various trenches and test pits. The position of the different furnace bases found during the excavations is also indicated, colored according to the chronological phase to which they belong (see Fig. 4 – Map A. adapted from Mayor et al., 2023; Photogrammetry P. Lamotte)

**Fig. 4:**
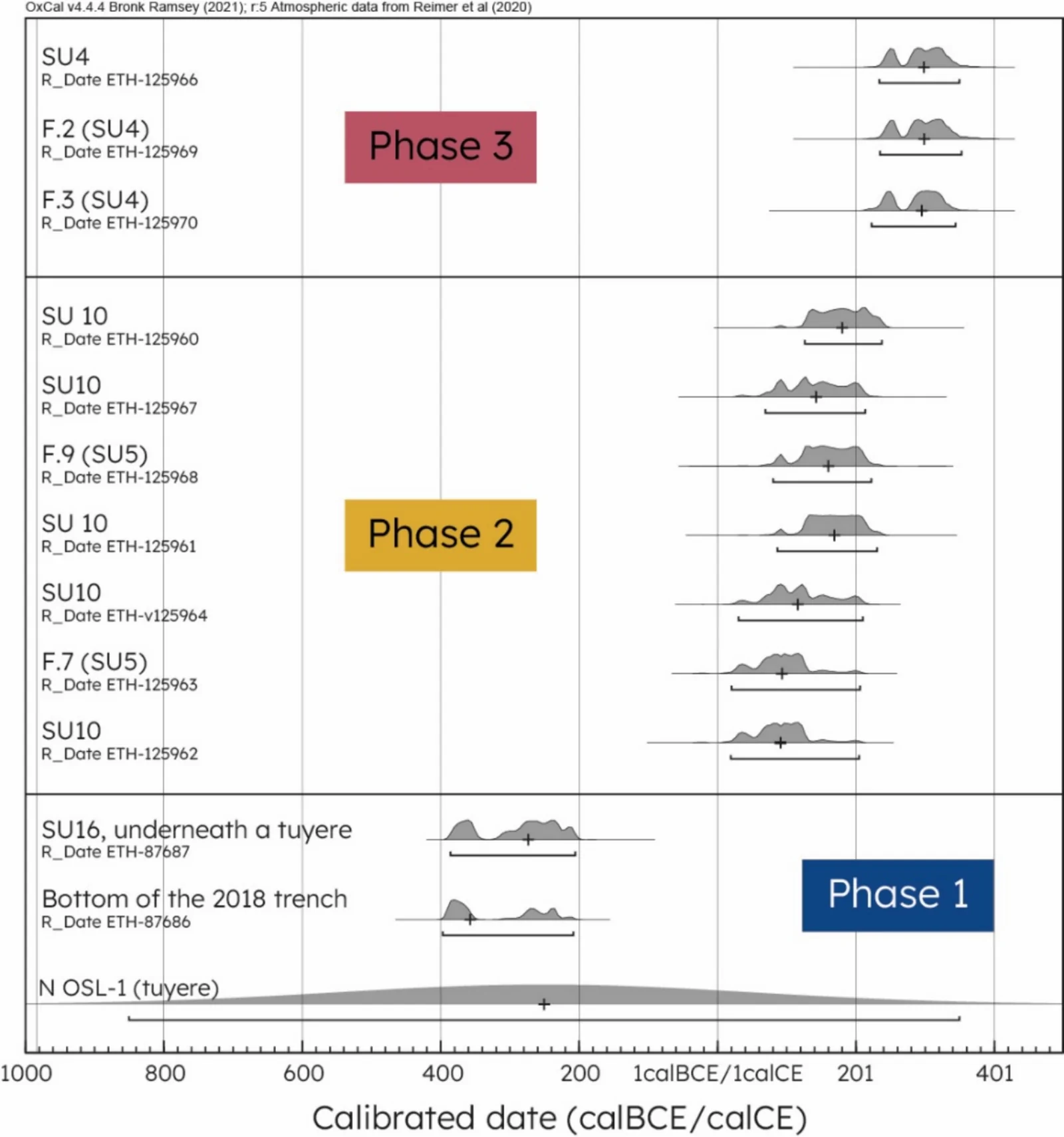
Results of radiocarbon dating on stratigraphically collected charcoal and OSL dating on a tuyerefrom DDW1 site (Adapted from Mayor et al., 2023)

At least three distinct phases of iron smelting have been identified (Figs. 3 and 4), revealing a progressive shift in furnace locations over time, from the central area of the slag heap toward its northern periphery (Mayor et al., 2023). The first phase, dating to the 4th–3rd centuries BCE, marks the earliest known iron smelting activity in the Falémé Valley. The second phase, active between the 1 st and 3rd centuries CE, represents a continuation of production with some technological or organizational adjustments. Finally, the third phase, dated to the 3rd–4th centuries CE, marks the latest phase of metallurgical activity recorded on the site. However, the chronological gap between Phase 1 and Phase 2 should not be interpreted as a period of site abandonment. The current dataset remains limited, and the excavation surface is too restricted to consider this apparent gap as a real hiatus. If the observed spatial shift in furnace locations reflects a chronological progression, it is possible that even earlier furnaces remain buried in the unexcavated southern half of the heap.

It should also be noted that no charcoal sample directly associated with the furnaces P1 has been dated. However, stratigraphic association shows that the layer from which samples ETH-87686 and ETH-87687 (Fig. 4) were taken corresponds to the same stratigraphic unit as the furnaces P1.

### Spatial Organization and Evolution of the Smelting Workshop DDW1

The DDW1 slag heap is roughly circular, measuring approximately 25 m from east to west and 22 m from north to south (Fig. 3). Excavations have revealed that the maximum thickness of the slag heap reaches 1 m. The total volume of the heap is estimated to be 200 m^3^. No furnace bases are visible on the periphery of the slag heap, which complicates our understanding of the spatial organization of the workshop.

**Fig. 5:**
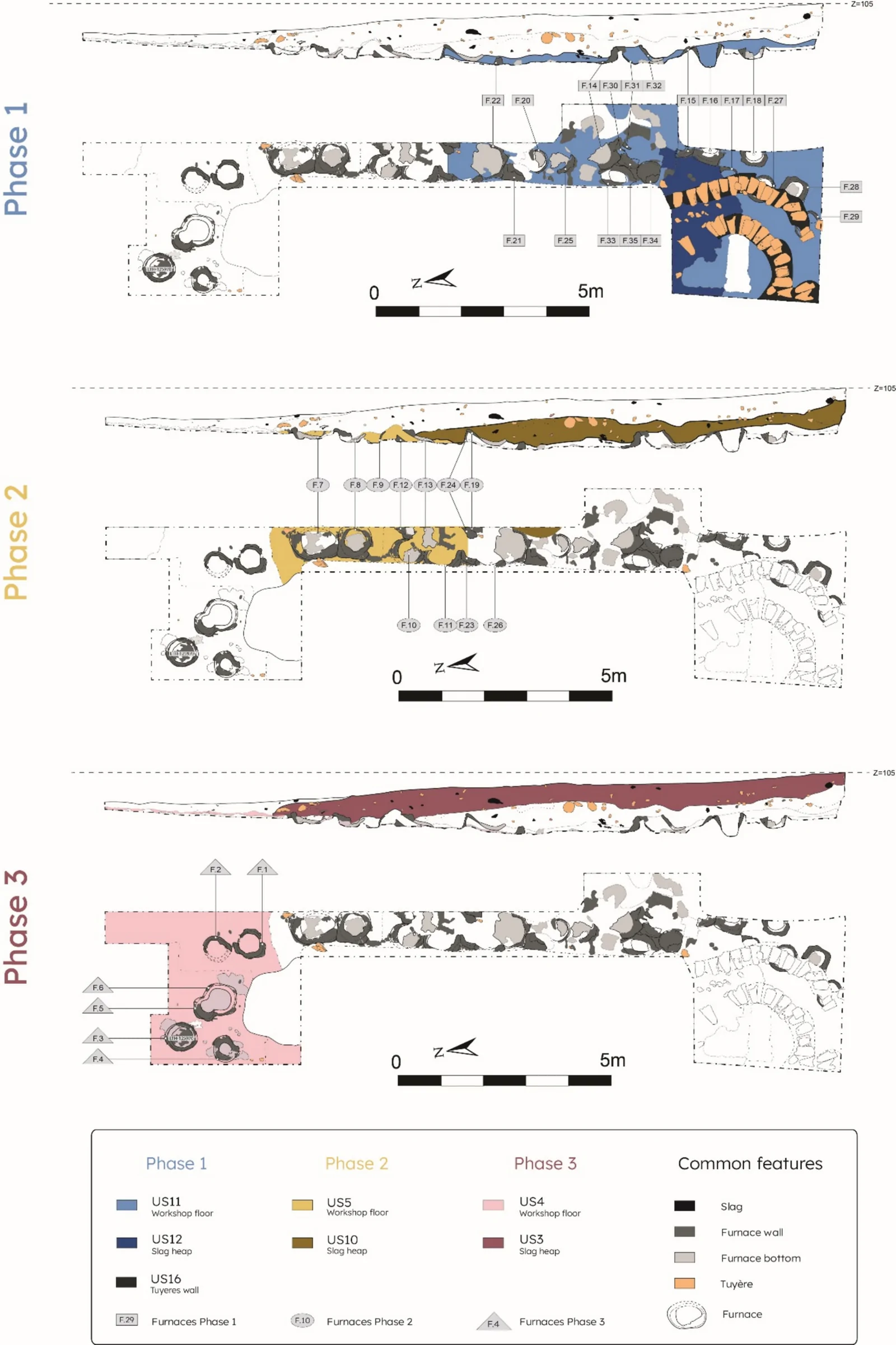
Simplified stratigraphic plan and cross-section of the test pits and trenches 4, 5, 8, and 2 of DDW1. It shows furnace bases according to the three chronological phases (P. Lamotte and M. Morel—for the complete description of the stratigraphy, see Mayor et al., 2023)

**Fig. 6:**
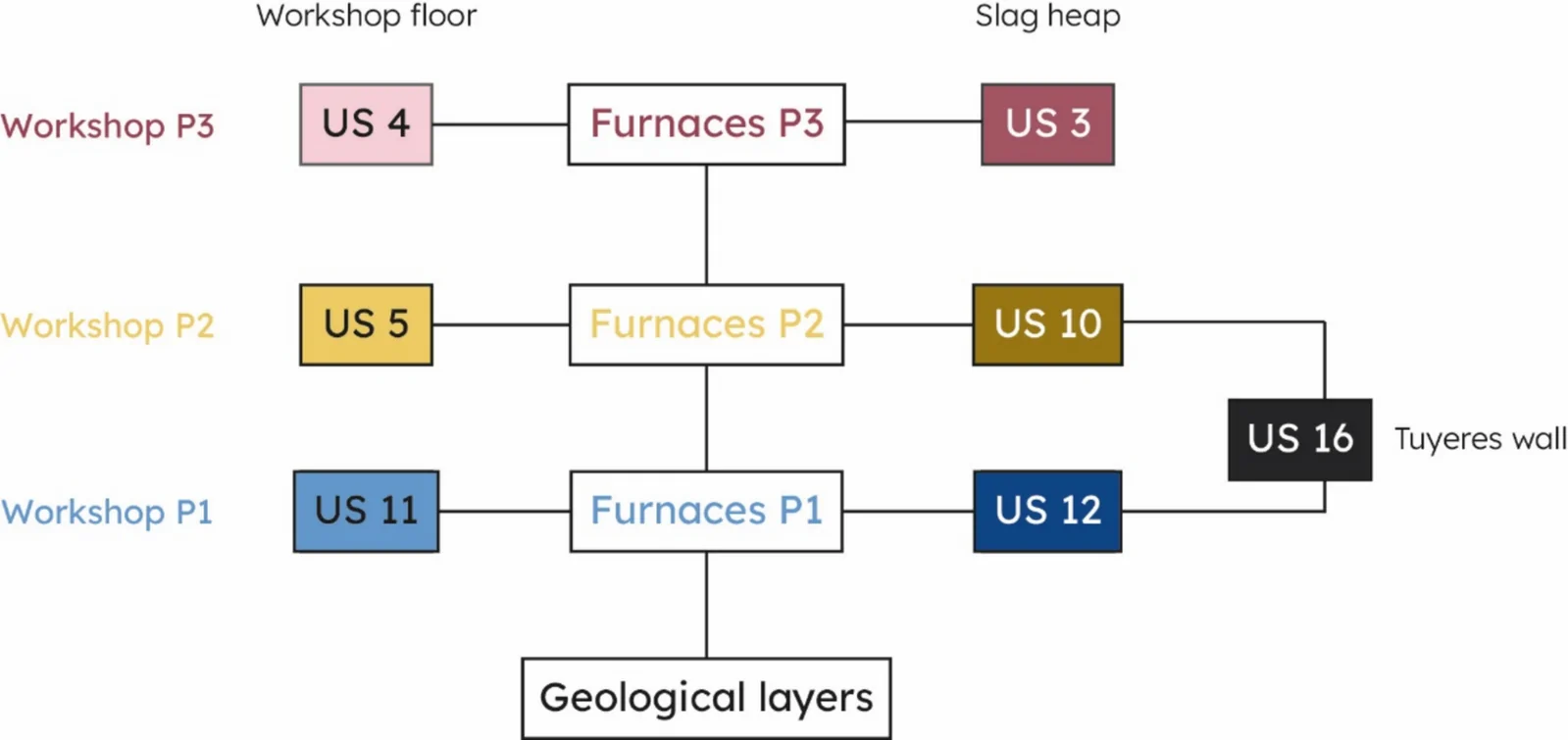
Simplified Harris matrix of the DDW1 stratigraphy (M. Morel)

During the second excavation at DDW1, the first trenches were dug at the periphery of the slag heap, following the example of previous excavations at Birandjikou, where furnaces had been identified in similar positions (Fig. 3 – Surveys 1, 3, 4, 6, 7; Mayor et al., 2023). Test pits 1, 3, 6, and 7 were negative and showed no trace of a smelting structure. However, this approach led to the discovery of furnace bases in pit 4 on the immediate northern periphery of the heap (Fig. 3). To further explore these findings, a 1-m-wide trench (Fig. 3—Trench 5) was conducted from Survey 4 to the center of the heap. This trench revealed a succession of 35 furnace bases along its entire length. Stratigraphic analysis identified three successive workshop floors (US11, US5, and US4) (Figs. 5 and 6), corresponding to at least three distinct phases of metallurgical activity.

The oldest phase (Phase 1) is marked by the presence of numerous furnaces (F.15, F.16, F.17, F.18, F.27, F.28, F.29, F.20, F.21, F.22, F.25, F.14, F.30, F.31, F.32, F.33, F.34, F.35). This phase may represent a significant stage in the accumulation of the slag heap, potentially reflecting repeated or prolonged smelting activity.

The second phase (Phase 2) includes furnaces F.7, F.8 (?), F.9, F.10, F.11, F.12, F.13, F.19, F.23, F.24, and F.26. These are associated with an accumulation of ironworking debris identified as US10.

Finally, the last phase (Phase 3) corresponds to furnaces F.1, F.2, F.3, F.4, F.5, F.6, and possibly F.8, and is linked to the metallurgical waste accumulation in US3. Further exploration of Survey 4 to the north confirmed that natural lateritic soil appears quickly beyond this point, ruling out the possibility of additional furnaces in that direction.

This excavation hence revealed a sequence of 35 furnaces, which overlap and intersect with each other. The working area gradually shifted toward the North over time, with newly accumulated metallurgical waste progressively covering earlier workspaces, ultimately forming the slag heap observed today.

### An Unusual Structure Made of Tuyeres

**Fig. 7:**
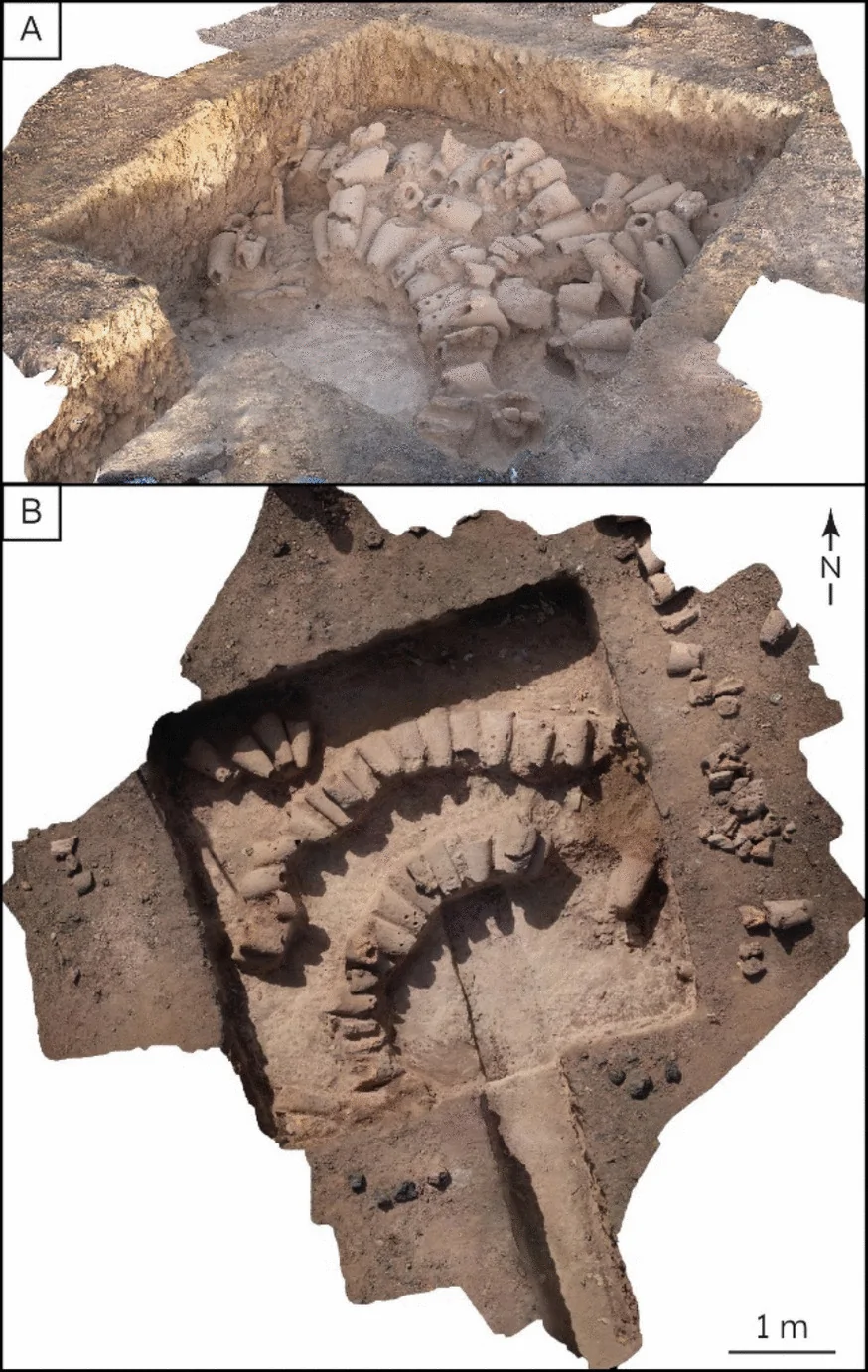
Photogrammetry of the stacked tuyere structure: **A** upper level with fragmented and displaced tuyeres; **B** lower level with complete tuyeres in situ. (**A** by A. Walmsley and D. Glauser and **B** from C. Ollier, 2018, p. 56)

During the first excavation in 2018, an unusual structure made of stacked used tuyeres was uncovered (Fig. 7). At the start of excavation, it appeared as a disordered pile (Fig. 7A), but progressive removal of sediment and pieces of displaced tuyeres revealed two arcs of carefully aligned tuyeres (Fig. 7B). The first layer of the outer arc comprises 18 tuyeres extending over c. 3 m, enclosing an inner arc of 16 tuyeres spanning c. 2 m. Although the original height of the feature cannot be determined, it likely consisted of at least two superimposed rows, with the upper row having collapsed.

All the tuyeres share the same morphology and dimensions (see Sect. "Morphology of the Tuyeres"). While some are broken, most are remarkably well preserved. Each shows traces of use but no evidence of repair, which suggests that they were intentionally stored, possibly for future reuse. Other functions cannot be excluded; however, in the absence of archaeological or ethnographic parallels, the precise purpose of this construction remains uncertain.

Designated as US16 (Figs. 5 and 6), this tuyere structure was found directly above the furnace bases of workshop P1. Its stratigraphic position raises the question of whether it belongs to period P1 or P2. A charcoal sample taken just beneath one tuyere (ETH-87687) yielded a date consistent with both the charcoal at the base of stratigraphic unit US11 (ETH-87686) and the OSL date obtained on a tuyere, linking this structure to Period 1. Spatially, it appears that the furnaces of P1 were first built and used, and that the tuyere structure was subsequently installed, still within the timeframe of period P1.

### Description of the Metallurgical Remains

#### Slag Morphologies

**Fig. 8:**
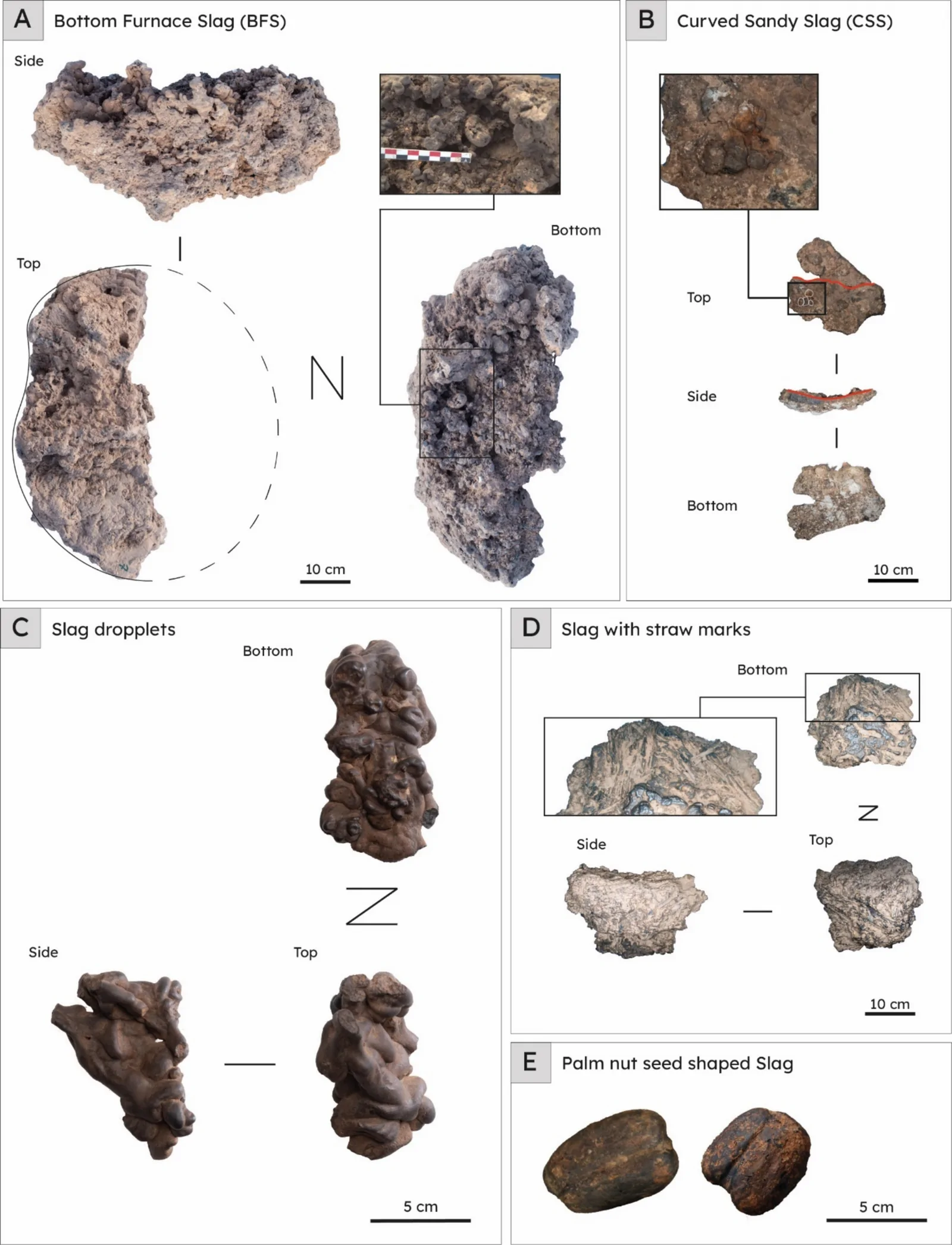
Illustration of the different slag morphologies found at Didé West 1 and characteristic of the technical tradition FAL02 (P. Lamotte and M. Morel)

There are very few slag pieces still in their primary position. Most of them are found in the heap, meaning in the waste disposal area. There is no obvious spatial organization of the slag within the heap based on their morphological characteristics, indicating no deliberate sorting by the smelters. Four morphological categories of slag were identified during the excavations (Fig. 8). These diverse morphologies are found in different proportions in the heap.

##### Bottom Furnace Slag

Bottom furnace slags (BFS) are massive internal slag cooling at the bottom of the furnace. They are circular or semicircular, measuring around 60 cm in diameter and about 10–20 cm thick, and their average weight for the complete pieces is 15 kg. They are slightly convex on their lower part as they mold the bottom of the furnace pit. They are mainly found as fragments in the DDW1 slag heap. Occasionally, clay-sandy fragments, corresponding to the building materials of the smelting hearth, are attached to it.

The BFS are fayalitic and constituted of dense grey material. They are made of an accumulation of many small cord-like droplets that compacted gradually throughout the smelting operation (Fig. 8A). Numerous small cavities can be observed, indicating that the slag droplets did not completely flow, probably because the temperature was not high enough in the bottom of the shaft.

Flow marks and slight depressions observed on the upper part of the complete slag pieces provide clues for reconstructing the tuyere placement.

The BFS pieces constitute most of the metallurgical waste and represent just over half of the total slag pieces (55%).

##### Curved Sandy Slag

The curved sandy slag (CSS) pieces are convex-shaped, typically with a thickness ranging from 1 to 4 cm and about 15 cm in length. This slag morphology is made of sandy clay material, sometimes with centimeter-sized ore fragments trapped in it (Fig. 8C). Moreover, fragments of partially smelted ore can sometimes be noticed on the upper surface of the CSS.

The CSS are formed when a sandy material sinters due to the high temperature within the furnace. Some parts clearly turned into liquid, with the formation of small flows and droplets indicating the orientation of the piece. These slag pieces are interpreted to be formed under the tuyeres, which were wedged in sandy material. This sand, located under the air inlets, which are the hottest points, melted to form the CSS. This slag morphology represents 20% of the slag assemblage.

##### Slag Droplets

The slag droplets are fragments of slag that have accumulated at the bottom of the furnace but have not fused with the BFS (Fig. 8B). They are therefore made of the same material as the BFS but are smaller in size, rarely exceeding 5 cm in width and a dozen centimeters in length. They represent about 15% of the total mass of slag.

##### Slag with Vegetation Marks

A small proportion of the slag pieces (approximately 10% of the total slag mass) exhibit distinctive vegetal imprints. Two main categories stand out. The first includes slag with grass straw marks, characterized by the parallel impressions of plant fibers (Fig. 8D). More unexpectedly, some slag fragments have taken the shape of *Borassus aethiopum* (palm tree) nut seed (Walmsley et al., 2020 – Fig. 8E). This type of slag morphology is unusual and has not been reported in other archaeological contexts.

These vegetal imprints are also observed on bottom furnace slag pieces. Straw and palm nuts were likely placed at the bottom of the furnace to create a porous support for the charge of ore and charcoal, ensuring space for slag accumulation. These are not combustion residues, as these organic materials would have been entirely burned before the slag even began to flow, leaving no visible archaeological traces. Similar practices have been documented in other West African metallurgical contexts, where furnaces were lined with plant material or, in some cases, even discarded tuyeres (Serneels et al., 2014).

#### Morphology of the Tuyeres

The tuyeres were made from a mix of orange clay with some sand and straw. Fracturing can be noticed on some pieces, suggesting tuyeres were likely made from material that was air-dried rather than pre-fired. During smelting, the clay undergoes dehydration due to high temperatures, resulting in the observed fractures.

**Fig. 9:**
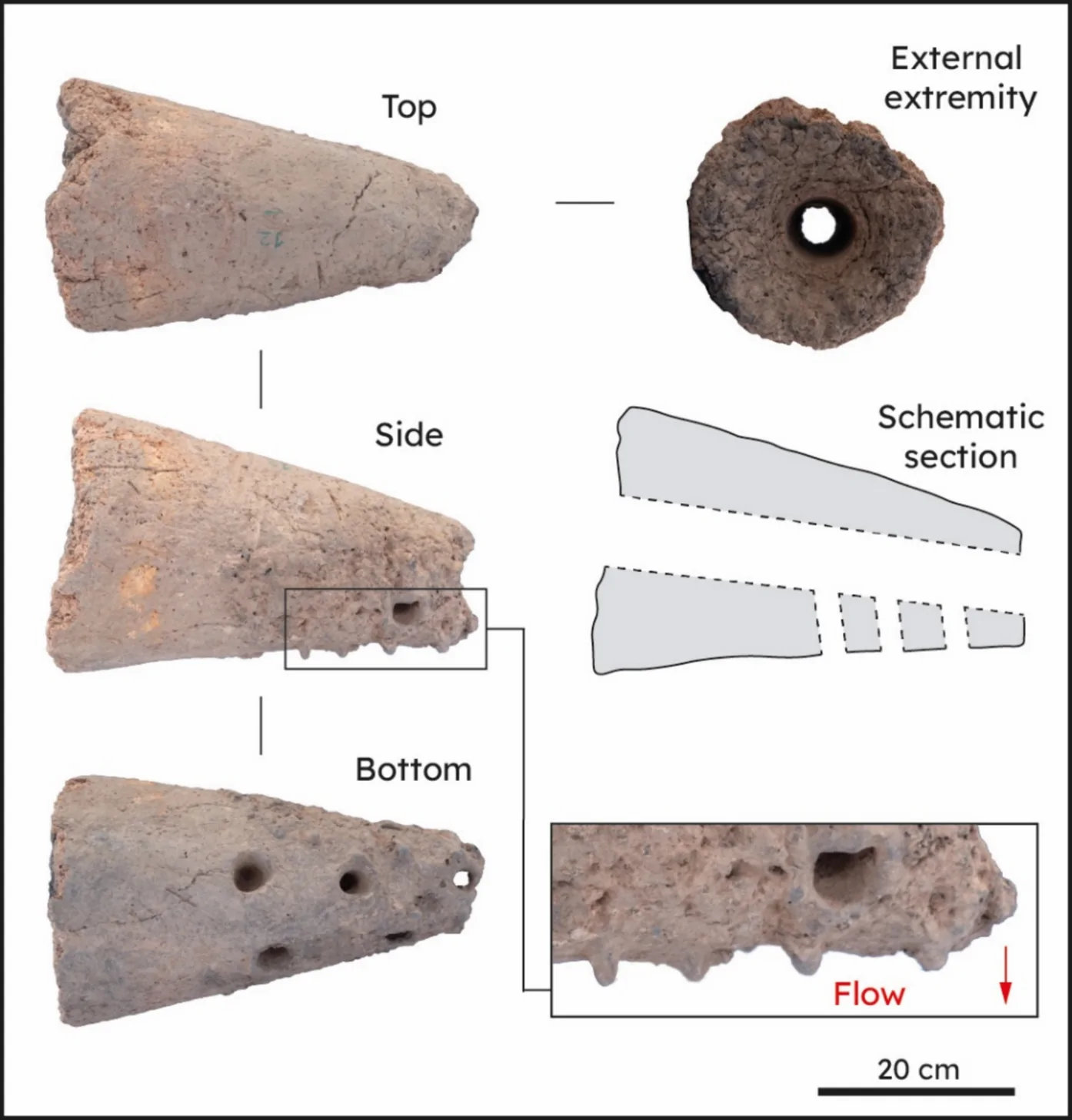
Illustration of a typical tuyere from Didé West 1 (P. Lamotte and M. Morel)

Tuyeres show a distinctive and very unusual morphology, a feature also observed at numerous sites of the Birandjikou sector (Fig. 1), and characteristic of this FAL02 technical tradition. The tuyeres are massive, measuring between 40 and 50 cm in length (Fig. 9). They have a conical shape, and the diameter of the extremity inside the furnace ranges from 10 to 15 cm, while the outer part measures about 20 to 25 cm. A 6-cm-diameter pipe pierces the cone lengthways. Additionally, the tuyere is perforated with 3 to 6 other holes measuring 3 cm in diameter, perpendicular to the primary pipe. The position of the holes along the tuyere is variable and does not seem to be standardized. They are likely positioned in the lower part of the tuyeres, as flows of melted materials can indicate the orientation of the piece. Some of these holes may also be partially filled by molten materials.

Given the large diameter of the internal pipe, these furnaces might initially be interpreted as relying on natural draft rather than bellows. However, several features such as the shift from a single tuyere in Phases 1 and 2 to possibly three tuyeres in Phase 3, as well as the existence of transversal perforations, suggest they operated with forced draft. These elements indicate that bellows were likely connected to the tuyeres.

The tuyeres extended about 20 cm into the furnace rather than being flush with the wall, as evidenced by tuyere fragments attached to the upper parts of the BFS. In our reconstruction of the smelting structure (Fig. 11), the transversal perforations appear to be positioned at ground level, approximately 20 cm above the bottom of the shaft. The function of these perforations remains unclear.

To explain the existence of sandy clay slag pieces, we believe the tuyeres were wedged in a layer of sand. Under the influence of heat, this sand, likely mixed with other materials such as ore fragments, would form these plano-convex slags, molding a portion of the underside of the tuyere.

### Smelting Furnaces

**Fig. 10:**
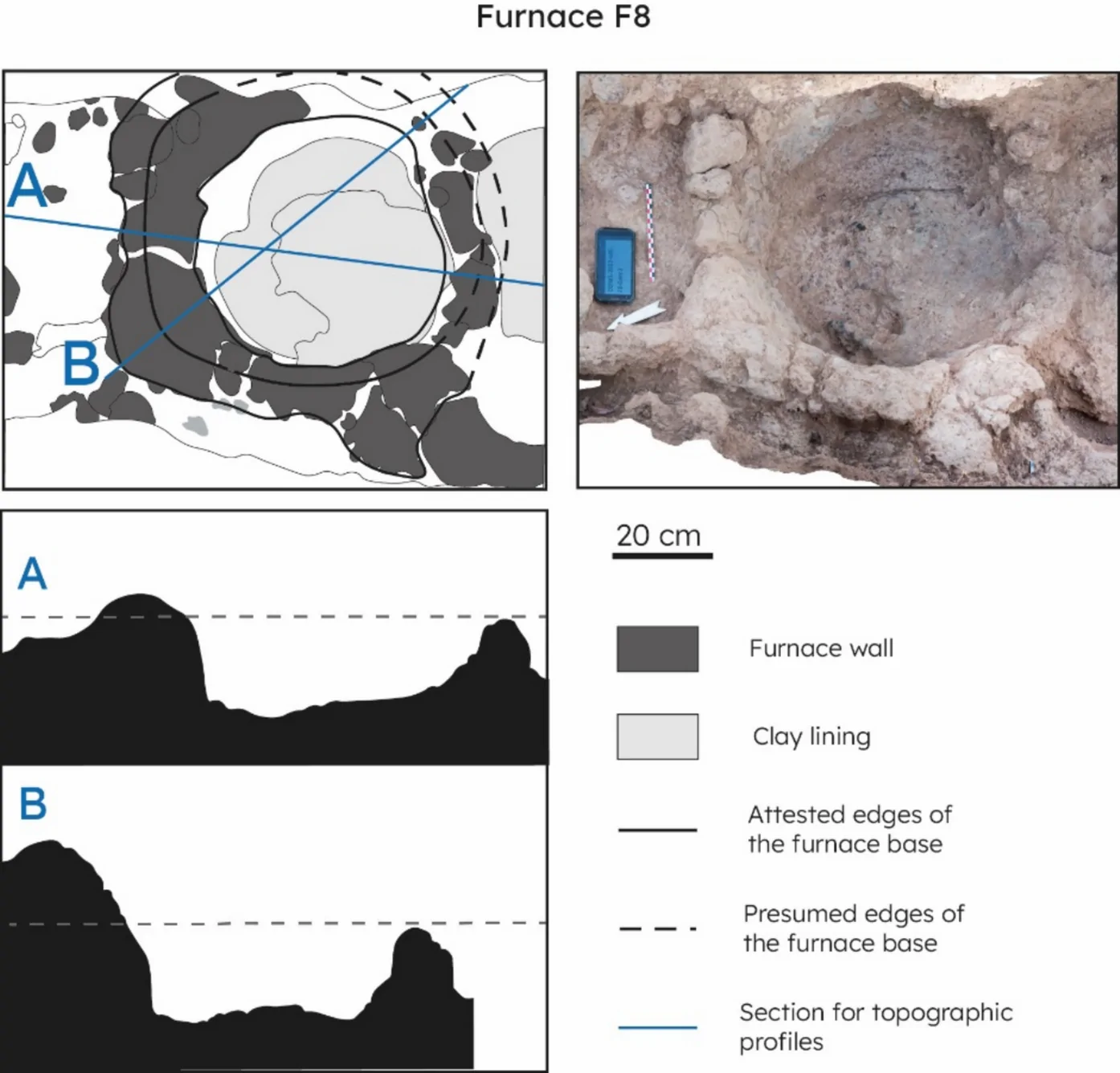
Drawing (upper left) and photogrammetric reconstruction (upper right) of furnace base F8. Profile sections of the furnace depth were generated from the photogrammetric model (lower left). (Photogrammetry: P. Lamotte, Figure: M. Morel)

As seen in the spatial organization of the DDW1 heap, excavations revealed a succession of furnace bases, with no preserved superstructures. These bases are cylindrical, ranging from 45 to 90 cm in diameter and 20 to 65 cm in depth (Table 1). They are bordered by a small clay rim (Fig. 10), which could mark the base of a former superstructure. This rim is not edged at ground level but reaches heights of up to 15 cm. It is probably not its original height, since they are found broken. No clear traces or arrangements indicate the placement of tuyeres or bellows.

**Table 1:** Table of the dimensions of the furnace bases recovered at DDW1. The dimensions were measured and calculated from the photogrammetry reconstructions

Furnace	Chronology	Perimeter (cm)	Diameter (cm)	Maximum depth (cm)	Volume of the shaft bottom (L)
F1	P3	193.5	60	27	76
F2	P3	165	52	20	42
F3	P3	198	63	31.7	99
F4	P3	159	47.5	23.6	42
F5	P3	144.5	46	31.2	52
F6	P3	218	70	33.7	130
F7	P2	290	89.5	NA	159
F8	P2	211	68.5	38	140
F9	P2	NA	50	30	59
F10	P2	202	64.5	34	111
F11	P2	247	79.5	50	244
F23	P2	NA	NA	31	NA
F24	P2	NA	NA	37	NA
F12	P2	194.68	62	23	69
F14	P1	182.12	58	35	92
F16	P1	229.22	73	54	226
F18	P1	188.4	60	31	88
F22	P1	135.02	70	24	92
F25	P1	257.48	82	39	206
F27	P1	197.82	63	18	56
F28	P1	178.98	57	42	107
F32	P1	NA	71	31	123
F33	P1	257.48	82	65	334
F34	P1	160.14	51	21	43

While slight variations in furnace base dimensions are observed across different chronological phases, their measurements (diameter, maximum depth, and estimated volume of the shaft bottom) provide only approximate indications of furnace size. The number of furnaces excavated and well-preserved for each phase is limited, and these measurements are highly dependent on preservation conditions. Some bases are partially cut by later furnaces, making it difficult to determine their original maximum diameter. Similarly, it is a well-known issue in archaeology that the preserved height of furnaces varies significantly, which directly affects depth measurements and calculated shaft volumes.

Phase 1 furnaces are built with a very pale clay, like the surrounding sediment. Ten well-preserved bases from this phase allowed for measurements, showing diameters between 51 and 82 cm and depths ranging from 18 to 65 cm. The estimated shaft volumes vary significantly, from 43 to 226 L (Table 1).

For Phases 2 and 3, a shift in construction materials (use of orange clay as lining) supports our interpretation of successive workshop phases. Despite this shift, Phase 2 furnaces maintain overall dimensions like those of Phase 1. Six well-preserved bases were measured, with diameters between 50 and 89 cm and depths between 23 and 50 cm. Their estimated shaft volumes range from 59 to 244 L, again showing high variability despite the small sample size (Table 1).

Phase 3 furnaces tend to be slightly smaller on average. The six well-preserved bases measured in this phase show diameters between 46 and 60 cm and depths between 20 and 34 cm, with estimated shaft volumes ranging from 42 to 130 L (Table 1). However, given the limited sample size, this trend may result from preservation biases rather than a deliberate technological change.

Overall, the furnace pits exhibit similar dimensions with minor variations. Some differences correlate with chronology, but they remain subtle and may be preservation related. For instance, Phase 1 furnaces are less well preserved, often partially destroyed by later stacked tuyere structures. While Phase 3 furnaces appear slightly smaller, the difference is marginal and not statistically significant.

## Discussion

### Reconstitution of the Smelting Technique

**Fig. 11:**
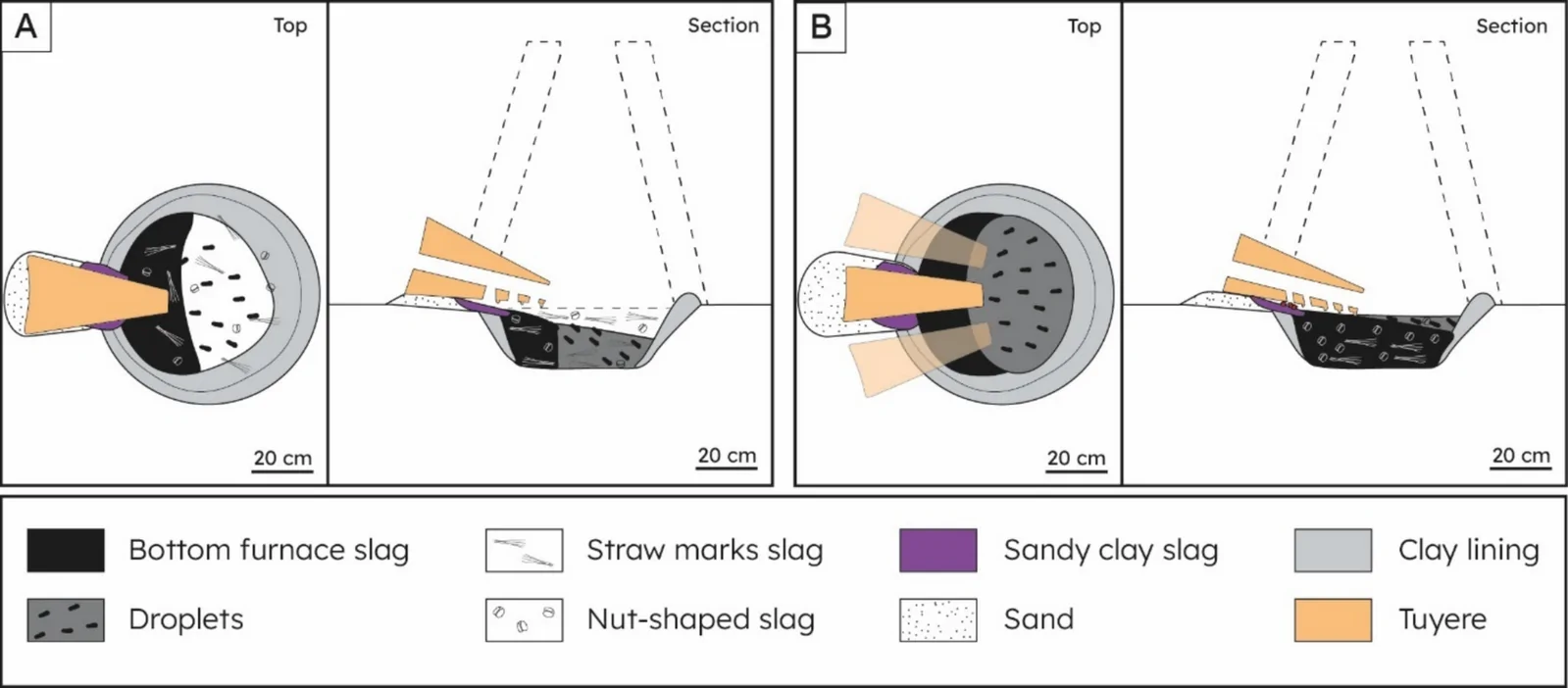
Theoretical reconstruction of the FAL02 furnace with the location of the different slag morphologies and tuyeres. **A** Reconstruction of the furnace for Phases 1 and 2 with a single tuyere. **B** Reconstruction of the furnace for Phase 3 with three tuyeres. It is not possible to reconstruct the exact shape and height of the superstructure (P. Lamotte)

Combining all observations made on the metallurgical remains from Didé West 1, particularly the detailed study of slag and tuyere morphology, allows us to propose a reconstruction of this iron smelting technique (Fig. 11).

Numerous furnace bases with clay lining were found, but no intact superstructure is preserved, except for small broken edges. Additionally, no wall fragments were recovered during the excavation. This situation is challenging to explain, and several hypotheses can be proposed even if none is entirely satisfactory:

- One possibility is that the absence of a superstructure is due to the use of bowl furnaces. This type of furnace has been documented in other regions of the world, particularly in Africa (Célis, 1989; Brown, 1995; Morel, 2023). However, bowl furnaces are generally expected to produce very heterogeneous slag and poorer reducing conditions compared to more confined environments (Morel, 2023; Morel & Serneels, 2021). Therefore, it seems unlikely that a smelting operation in an open pit would have produced the large slag pieces observed at DDW1. Moreover, preliminary chemical analysis of slag from this technical tradition at Birandjikou suggests that smelting conditions in these furnaces were relatively good. Indeed, a significant proportion of iron oxides were successfully reduced to metal, with minimal iron remaining in the Fe^3+^ oxidation state (Walmsley et al., 2020). These results contradict what would typically be expected from a bowl furnace.
- A second possibility is that the superstructure was not properly fired and thus did not preserve well. The hypothesis of a permanent, immobile superstructure that was destroyed and left no fragments in the archaeological layers is unconvincing.
- Lastly, it is plausible that the superstructure was removable, as seen in other ethnographic (Echard et al., 1983) or archaeological contexts (Serneels et al., 2012). In this case, the superstructure would have been removed after each smelting operation to recover the metal and empty the slag from the bottom of the furnace. It might have been relocated each time a new furnace base was used. These removable superstructures could have been reused multiple times, which would account for the absence of wall fragments found. It is likely that the superstructure was not made from organic material; otherwise, we would expect to find vegetal impressions on the furnace rims. However, the complete absence of wall fragments remains puzzling, as the superstructure would have had to be properly fired to ensure its mobility.

At the edge of the furnace pit, set in sand, one to three tuyeres are positioned. They are inserted about 20 cm into the furnace and pointing slightly downwards. The extremity of the tuyere is close to the center of the hearth of the furnace. There are no marks on the tuyeres indicating a significant temperature difference between the part of the tuyere located inside the furnace and the part outside. Instead, there appears to be a gradual impact of the temperature. Therefore, the tuyere is probably not sealed in the superstructure, with clay, for example, but rather positioned in a large opening. For phase 3, where multiple tuyeres seem to be used, we can see that they are placed next to each other, on only one side of the furnace, rather than around it.

Ore, likely in the form of laterite grains (Fig. 2), was loaded in the furnace with charcoal used as fuel. Grass straw, as indicated by the imprints on the slag, and, more unexpectedly, palm nuts, were placed at the bottom of the furnace to facilitate the flow of slag during the smelting process.

During the smelting process, fayalitic slag initially forms as small droplets that flow and accumulate at the bottom of the furnace. If the temperature is high enough, these droplets coalesce, eventually forming a large, compact BFS that fills the furnace pit. The higher the temperature, the more the droplets can merge into a homogeneous block. Conversely, if the temperature is insufficient, the droplets remain separate and do not fully fuse, resulting in numerous independent slag fragments.

This difference in slag formation is particularly obvious when comparing the different phases. In Phases 1 and 2, BFS often takes the shape of a crescent and does not completely fill the furnace pit. These incomplete slag blocks are systematically associated with numerous independent slag droplets, indicating that the temperature was not always high enough for full coalescence (Fig. 11A). In contrast, during Phase 3, BFS is typically found as a single, compact mass with a circular base, completely filling the furnace pit. This suggests that all slag droplets were sufficiently heated to merge into a homogeneous block (Fig. 11B).

These smelting structures, like those identified in the sector of Birandjikou (Mayor et al., 2022), form a compact and dense spatial arrangement. Many furnace bases overlap or cut into earlier ones, showing that they were built successively rather than used at the same time. The evidence suggests that a single furnace was active during each smelting episode, after which it was emptied and reused multiple times before being replaced by a new one.

### Variability and Standardization of the Smelting Remains

Iron smelting at Didé West 1 extended over around 800 years (Fig. 4), divided into three phases based on radiocarbon and stratigraphic data. Despite this long timeframe, the FAL02 smelting tradition remained largely unchanged, reflecting a long-standing technical continuity.

Indeed, the main technical features are consistent over time. The FAL02 tradition is characterized by small-diameter circular furnaces, with tuyeres positioned side by side on one side of the furnace. These tuyeres exhibit a particularly distinctive shape with transversal perforations. The slag management system relied on internal flow, with no evidence of external tapping. Instead, slag was systematically removed from the bottom of the furnace after each smelt. These technical features are consistently observed across all three chronological phases, leaving no doubt about the technological continuity at DDW1. Moreover, the remains associated with the FAL02 tradition, both at DDW1 and Birandjikou (Fig. 1), differ significantly from those linked to other metallurgical techniques in the Falémé Valley, like FAL03 (group 3 in Walmsley et al., 2020) and from those documented elsewhere in northeastern Senegal, mostly later (Bocoum 2002; Deme, 2003; Killick, 2016; Dianifaba, 2020), where furnaces were typically single use.

On the other hand, minor variations can be observed within the FAL02 technique along the chronology. The furnace bases that were preserved display some variability. Diameters and depths vary by several centimeters, directly affecting the perimeter and volume of the hearths. Although these dimensions remain broadly consistent—especially when compared with other technical traditions—Phase 3 bases tend to be slightly smaller. Still, no clear evidence points to a significant chronological evolution. These variations suggest more probably that the technique was not rigidly standardized but instead adapted to each individual furnace. Yet caution is required: the poor preservation of the bases makes it impossible to reliably reconstruct their original height.

**Fig. 12:**
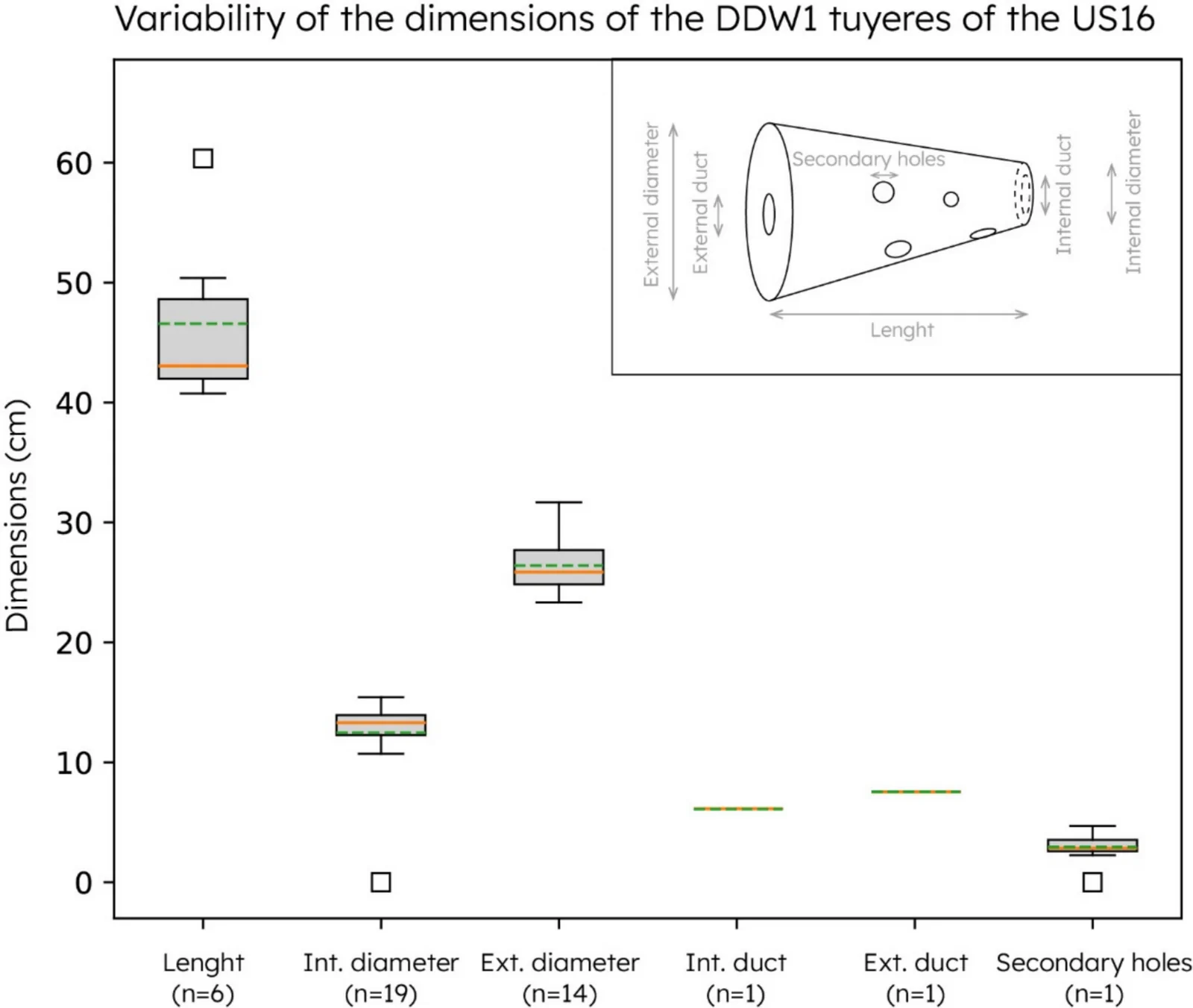
Boxplot showing the variability of the dimensions of the DDW1 tuyeres. All the measured tuyeres (*n* = 28) were collected from the semicircle structure, and all the dimensions were measured from photogrammetry 3D models. Because most of the collected tuyeres were partially broken, all the dimensions could not be measured on every sample (M. Morel)

As for tuyere use, Phases 1 and 2 likely involved a single tuyere, not always positioned at the center of the BFS (Fig. 11A). In Phase 3, by contrast, evidence from the upper surface of BFS suggests three tuyeres arranged side by side (Fig. 12B). Whether this change influenced the design of the furnace superstructure remains uncertain.

DDW1 tuyeres exhibit only minor variations. Measurements on 30 complete tuyeres from the semicircular structure (US16 – see Figs. 5 and 7) show highly consistent dimensions (Fig. 12). However, the number of transversal perforations varies between three and six per tuyere. These holes are not always in the exact same position along the tuyere, indicating a lack of standardization in manufacturing, though they are consistently oriented downward. Unfortunately, data on tuyeres from all the phases are unavailable, preventing a full assessment of whether this standardization persisted or evolved over time on this site.

Finally, subtle differences emerge when comparing the slag assemblage across chronological phases. The BFS pieces from Phases 1 and 2 only partially filled the hearth, unlike those from Phase 3, which are larger and completely filled the hearth. The BFS from Phase 3 is also more compact, whereas those from Phases 1 and 2 look more like a loosely packed agglomerate of droplets. This shift suggests a minor evolution in technical practices during Phase 3. However, due to a lack of comprehensive data on slag assemblages, it remains uncertain whether these larger BFS correlate with fewer slag droplets or other changes in other slag morphology.

The superstructure, unfortunately, cannot be discussed: no evidence indicates either continuity or change over time, nor any clear standardization from one smelt to another within the same phase. Since no wall fragments were recovered from any phase, major structural changes are unlikely.

The minor evolutions observed over time come as no surprise given the site’s extensive period of use. The observed technical consistency over such a prolonged period is therefore remarkable. Rather than drastic technological shifts, the data suggest knowledge transmission and gradual adjustments, allowing for slight variations without disrupting the overall method. This enduring stability prompts important questions: Could it be explained by limited interaction with other metallurgists’ communities active in the same time in the area or by conscious maintenance of a distinctive cultural trait? Does it reflect efficiency—whether economic or technical—of the method, in comparison to other ones? (Morel et al. in preparation).

### Duration and Intensity of Smelting Activities

**Fig. 13:**
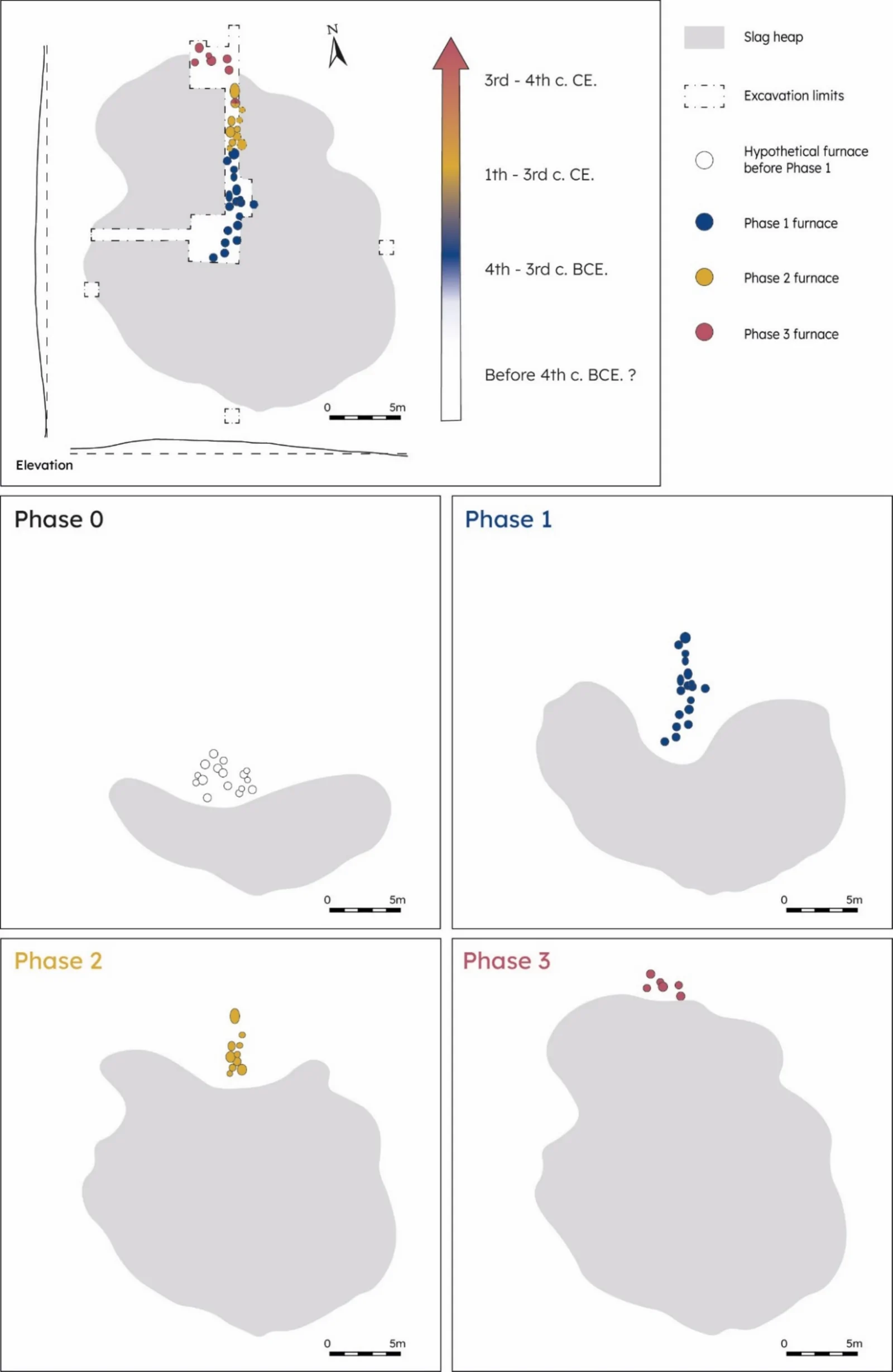
Hypothetical reconstruction of the spatial development of the DDW1 heap. (Top) General plan of the heap showing excavation areas and excavated furnace bases, color-coded according to their chronological attribution. (Bottom) Series of schematic reconstructions illustrating the spatial evolution of the workshop over time. Phase P0 represents a hypothetical stage predating Phase 1. The distribution of furnace bases suggests a progressive northward shift of smelting activities as the heap expanded (M. Morel)

Stratigraphic analysis and radiocarbon dating show the existence of at least three distinct smelting phases, reflecting a clear chronological and spatial progression of the metallurgical activity from the center to the north of the heap (Fig. 13—Mayor et al., 2023). The deposition of metallurgical waste followed this same pattern, gradually shifting northward along with the furnaces (Figs. 5 and 13).

This sequence suggests a long-lasting metallurgical tradition, with production spanning at least 800 years (Fig. 4). Phase 1 appears to date between the 4th and 3rd centuries BCE, followed by Phase 2 between the 1 st and 3rd centuries CE, and finally Phase 3 between the 3rd and 4th centuries CE. The southern half of the heap has not yet been excavated, and given the observed northward shift of furnaces over time, it is possible that even older furnaces remain undiscovered in this area (Fig. 13).

The wide distribution of ^14^C dates, spanning nearly eight centuries, does not necessarily imply continuous activity over this entire period. If smelting had been ongoing without interruption, the estimated number of operations per year would be especially low. Instead, the evidence suggests a pattern of intermittent activity, with phases of use followed by periods of abandonment. This interpretation is reinforced by the estimation of the total number of smelting operations conducted at DDW1 and the reflection on how many times a furnace has been used.

To estimate the number of smelts conducted in one single furnace, we sorted the metallurgical waste according to their morphology and weighed them (Table 2). The average mass of slag of each morphology per cubic meter is calculated (Robion-Brunner & Serneels, 2017). A bottom furnace slag corresponds to a single smelting operation. Estimating the number of BFS gives us the number of smelts carried out at Didé West 1.

**Table 2:** Mass of slag by morphology measured in the DDW1 slag heap and estimation of the number of smelting operations

	Mass per m^3^(kg/m^3^)	Mass in the DDW1 heap (kg)	Number of smelts (for 15 kg of slag/smelt)
(a) BFS	109.5	21,900	
(b) Straw marks	49.75	9950	
(c) Nut shaped	7.25	1450	
(d) Droplets	114.25	22,850	
(e) Sandy clay	111	22,200	
*Total a* + *b* + *c*	166.5	33,300	**2220**
*Total a* + *b* + *c* + *d*	280.75	56,150	**3743**
**Total slag**	391.75	78,350	
Tuyères	119.5	23,900	
**Total waste**	511.25	102,250	

In our calculations, we included slag fragments classified as “straw marks” and “nut-shaped” within the BFS category, as they are indeed broken pieces of BFS. Based on this classification, we estimate that the DDW1 heap contains approximately 166.5 kg of BFS fragments per cubic meter. Given that the mound has a total volume of 200 m^3^, this corresponds to an estimated 33 tonnes of BFS.

Based on well preserved pieces, the average mass of a single BFS is estimated at about 15 kg. This suggests that approximately 2200 BFS were produced at Didé West 1, implying that about 2200 smelting operations were conducted. This first estimate assumes that droplets are considered a byproduct, meaning that each smelting operation would have produced one BFS weighing 15 kg, along with an estimated 10 kg of droplets.

Alternatively, if the 22′850 kg of droplets in the DDW1 heap are interpreted as unconsolidated BFS rather than a separate byproduct, the total number of smelting operations could be as high as 3740. The actual number of smelting operations at DDW1 likely falls between these two estimates, ranging from about 2200 to 3800.

We now seek to estimate how many times each furnace at DDW1 has been used on average. A total of 35 furnace bases have been uncovered, meaning that a minimum of 35 furnaces existed. However, as we have only excavated a small portion of the heap, additional furnaces remain undiscovered. While it is difficult to speculate on data that is not available, a reasonable estimate suggests 100 furnaces. For a high-end estimate, we might consider a maximum of 200 furnaces.

Using these estimates, each furnace would have been used at least around 10 times, with an upper estimate of 109 times (Table 3). However, this maximum value appears unrealistic given the nature of the furnace remains, which appear to be relatively fragile structures, not designed for long-term, continuous use but rather for intermittent, short-term use. It is more likely that each furnace was operated for one or two dozen smelting operations before being abandoned and replaced by a new one.

**Table 3:** Number of smelting operations that were conducted in a single furnace depending on the estimated number of furnaces at DDW1 and on the total number of smelting operations conducted over the 800 years of activity

	Estimated number of furnaces	Minimum number of smelting operations (2200)	Maximum number of smelting operations (3800)
Number of smelting operations per furnace	35	63	109
	100	22	38
	200	11	19

To estimate the frequency of smelting operations per year, we know that the metallurgical activity at DDW1 lasted approximately 800 years (Fig. 4). Given that between 2200 and 3800 smelting operations took place at the DDW1, this equates to an average of about 3 to 5 smelting operations per year. This is relatively low compared to other mass production metallurgical districts described in other regions of West Africa (e.g., Robion-Brunner et al., 2006; Perret & Serneels, 2009; de Barros, 2021; Morel et al., 2025).

The idea that each furnace was used dozens of times over multiple years, while only conducting 3 to 5 operations annually, is not convincing. The furnace bases found at DDW1 do not appear to be structures that could be abandoned for weeks or months and then reused. It is more likely that a furnace was used for one or two dozen of successive smelts before being abandoned. A new one was then built nearby when iron production resumed. This suggests a system of intermittent production campaigns rather than continuous, large-scale production. The site was likely used during short periods of time and/or abandoned for several months or years between production phases, with iron being produced only when needed.

How, then, was production organized to match the number of smelting operations calculated over 800 years? Experimental archaeology suggests that small furnaces like those found at DDW1 require around ten people per day to gather ore, produce charcoal, and conduct smelting (Crew, 1991). Ethnographic and historical sources provide examples of both intensive and less intensive production strategies. In some cases, such as India (Buchanan, 1807) or central Madagascar in the 20th century (Breton, 1898; Morel, 2023), two to three smelting operations were conducted in a single day. Conversely, in other contexts, such as Burundi (Celis, 1989) and Kenya (Brown, 1995), only one smelting operation was carried out in one day. In either case, achieving the previously estimated 20 to 40 smelting operations per furnace base within a year would have been feasible, likely taking a few weeks of work by a small group of people. Given the seasonal nature of agricultural cycles, it would have been feasible to schedule iron production during times of reduced agricultural labor demands. This model suggests that iron smelting was not a full-time occupation for specialized artisans but rather a secondary activity conducted intermittently, probably seasonally.

However, DDW1 is not the only slag heap in the region, and the absence of production at this workshop in certain years does not imply a regional interruption of iron smelting. Dozens of other sites belonging to the same FAL02 technical tradition have been identified, and production may well have continued there when DDW1 was inactive. To fully understand the site, it is therefore important to consider the broader metallurgical landscape, both in the Didé area and across the wider Birandjikou district (Fig. 1). Several nearby sites have been excavated and dated, showing identical technical features and variability, corresponding to the Phases 2 and 3 recognized at Didé West 1, as well as a later Phase 4 (Mayor et al., 2025). These sites may have been used alternately, especially in the context of relatively mobile populations.

In an agropastoral economy, the mobility of populations, or even of entire villages, is not unexpected. Although drawing direct parallels between contemporary communities and those that lived 2500 years ago remains tenuous, oral surveys conducted by A. Mayor and N. S. Guèye in the Falémé valley reveal a high degree of village mobility today. Modern agropastoralists move frequently, often relocating their settlements, not over great distances, but repeatedly. This mobility is due to recurrent fires, water scarcity, flooding, land exhaustion, or demographic pressure (Huysecom et al., 2013). It would therefore be unsurprising to find a similar pattern of mobility among the ancient villages where metallurgists of the FAL02 tradition were living. Moreover, the movement of metallurgical workshops may have been driven by resource depletion, such as the exhaustion of charcoal supplies or ore deposits (Pelet, 1974; Iles, 2016).

Hence, a better understanding of population dynamics, land use, and variability in the intensity of metallurgical production would be possible through a comprehensive examination of all slag heaps in the region, as well as settlement sites, which still have to be identified.

### Originality of the FAL02 Technique

The quality of the data collected as part of this study on DDW1 allows us to question some of the technical specificities of FAL02.

One of the most surprising aspects of this technique, given its early chronology, is the reconstruction of a forced-draft system, meaning that bellows were used. This represents one of the oldest known instances of forced-draft furnaces in African iron metallurgy. However, in many other sites, poor preservation prevents any clear reconstruction of the air supply system, as their remains are often limited to furnace bases. The ability to infer this feature at Didé West 1 is therefore a rare and significant contribution to the understanding of early smelting technologies.

Beyond the forced-draft system, another remarkable feature of this tradition is the highly unusual tuyere design. Archaeological, ethnographic, and historical data point to only two other known cases of tuyeres with transversal ducts, both from Ivory Coast: the KAN4 tradition (active between the 11th and 17th centuries CE; Serneels et al., 2013) and the VAN2 technique (11th–13th centuries CE; Yeo Arouna, SAfA conference, 2025), though insufficient chronological data limit precise dating. The extent of technical similarities between FAL02 and these Ivorian traditions remains unclear and very far in time, but the presence of perforated tuyeres in both regions raises the possibility of technological transfers. The striking resemblance between the tuyeres at Didé West 1 and those from Ivory Coast (Serneels et al., 2013) may suggest shared technical knowledge that merits further investigation.

These transversal perforations on tuyeres are not a common feature in West African iron metallurgy, nor do they present an obvious technical advantage that would have led to their widespread diffusion. This raises the question of whether they served a functional purpose or were a distinctive cultural trait of the FAL02 tradition. Their consistent presence suggests that they had a specific role in furnace operation. One possibility is that these openings modified the airflow dynamics within the furnace. If air was expelled through multiple outlets, particularly in a downward direction, it could have altered the volume and shape of the high-temperature zone. Rather than concentrating heat at the tuyere’s main outlet, the airflow may have been more dispersed. Experimental tests on tuyeres conducted with Robert Seiler (Mayor et al., 2025) confirm that such tuyeres may have created a localized hot zone beneath the tuyere.

Determining the precise function of these perforations will require further experimental and comparative studies. However, a key question remains: why would smelters have sought to generate additional heat lower in the furnace? One hypothesis is that this design was intended to enhance the separation of slag and metal, possibly to compensate for insufficient temperatures at the bottom of the shaft. This idea is supported by the structure of the BFS pieces, which are not constituted of dense and fully molten slag, but of an aggregate of small droplets. Such a slag texture suggests that slag was not completely liquid and that smelters may have struggled to maintain a high temperature at the furnace bottom. It could have affected the efficiency of slag drainage and metal recovery. If this was indeed a recurrent issue, the perforated tuyere design might have been a deliberate adaptation to improve the smelting process.

This tuyere design was not the only possible solution to temperature regulation within the furnace. The smelters could have opted for alternative strategies, such as constructing shallower furnace pits or increasing the number of tuyeres. It remains uncertain whether this design had unintended consequences, such as reducing the overall yield due to restricted airflow at the main tuyere outlet, which should have lowered the temperature in this part of the shaft.

However, it is important to consider that once a functional solution is established, optimization is not always a priority, especially if production levels are sufficient to meet the needs of the local community. Rather than striving for maximum efficiency as in our modern industrial practices, where optimization and increased output are primary goals, early smelters may have prioritized stability and reliability. In many precolonial African contexts, technological choices were not solely dictated by economic productivity but were also shaped by cultural considerations (Montes-Landa et al., 2024). The persistence of a process that was “good enough” for their specific needs suggests that innovation is not the only factor driving the longevity of a technique—sometimes, its mere functionality in a culturally and economically stable context is enough to ensure its continuity.

## Conclusion

The investigations at Didé West 1 extend far beyond the documentation of excavated remains or the description of smelting features. They provide critical insights into the organization of production and the long-term evolution of the metallurgical tradition called FAL02. High-quality fieldwork has revealed an exceptionally well-preserved technique, including 35 furnace bases spanning nearly 800 years and a remarkable arrangement of used tuyeres forming semicircular alignments. The FAL02 tradition can thus be characterized by small circular furnaces and unusual transversally perforated tuyeres. Lateritic ores were loaded into the furnace and slag accumulated at the bottom of the shaft rather than being tapped externally. Another unusual trait is the use of palm nuts as stuffing material, a practice so far unique in the archaeological record. The nature and presence of a superstructure remain uncertain, but available evidence makes the existence of bowl furnaces unlikely.

The site of DDW1 is also remarkable for its chronological span, covering nearly 800 years of activity. Even more striking is the stability of the FAL02 tradition throughout this period, with only minor variations observed. Further excavations, particularly to the south of the heap, could clarify both the spatial extent of the workshop and its earliest phases.

Unlike the large-scale production systems documented in some later West African districts, smelting at DDW1 was likely intermittent and seasonal, probably limited to only a few operations per year, with possible interruptions over several years if iron was not in immediate demand. Such relatively small-scale production was nevertheless sufficient to sustain metallurgical know-how over the long term without major technical changes. The persistence of this practice suggests a technique that was well adapted to local needs and did not require significant modifications. Its limited intensity also implies a relatively low impact on resources, particularly fuelwood, and on the surrounding environment. Although anthracological studies were not carried out due to the lack of charcoals, the extraordinary longevity of this metallurgical tradition supports this hypothesis.

The FAL02 tradition is not confined to DDW1. Numerous slag heaps associated with the FAL02 technique have been documented at Birandjikou and Tiéyé. Although these sites lack the deep chronology of DDW1, they indicate that several workshops may have been active simultaneously and that this tradition seems to last until the 8th century CE, meaning a total duration of at least a millennium. The overall scale and rhythm of production across the district, however, remain to be fully calculated (Morel et al. in prep). Furthermore, FAL02 coexisted with other distinct metallurgical traditions in the Falémé Valley, such as FAL01 at Madina Cheikh Oumar 1 (4th–3rd century BCE), FAL03 at Madina Cheikh Oumar 2 and Dakaba (4th–7th century CE), and FAL04 at Didé West 3 (5th–6th century CE) (Walmsley et al., 2020; Mayor et al., 2025). This diversity reflects a dynamic and complex metallurgical landscape that still needs to be better understood. A comparative study of associated pottery will bring new light on the cultural spheres to which belong the different iron producers and on the exchanges between sites and traditions (Mayor et al. 2025).

At a broader scale, meaningful comparisons between FAL02 and other West African traditions remain limited by the lack of well-dated early sites. One of the few comparable traits is the presence of perforated tuyeres, paralleled only by later traditions in Ivory Coast (KAN4 and VAN2, second millennium CE). In contrast, the use of palm nuts as furnace stuffing material appears to be a unique innovation. Other features, such as the use of slag-pit furnaces, are not distinctive enough to establish direct technical lineages. As is often the case, additional excavations and surveys are needed to refine our understanding of regional connections and to better situate FAL02 within the broader history of African iron metallurgy.

## Data Availability

All data generated or analyzed during this study is included in this article. Additional information, if required, can be found in the annual reports of the foundation Schweizerisch-Liechtensteinische Stiftung für Archäologische Forschungen im Ausland (FSLA), which are publicly accessible (Mayor et al., 2018, 2019, 2020, 2021, 2022). Any other data not included in the article is available from the corresponding authors upon reasonable request.

## References

[CR1] Alpern, S. (2005). Did they or didn’t they invent it? Iron in Sub-Saharan Africa. *History in Africa,**32*, 41–94.

[CR2] Arouna, Y. (2025). Following the road of the KAN 4 technical tradition. *Oral presentation at the 27th Biennial Meeting of the Society of Africanist Archaeologists, Faro, Portugal*.

[CR3] Bocoum, H. (dir.). (2002). *Aux origines de la métallurgie du fer en Afrique : une ancienneté méconnue; Afrique de l’Ouest et Afrique centrale*. Éd. Unesco (« Mémoire des peuples »).

[CR4] Breton, A. (1898). Fabrication du fer en Emyrne. *Notes, Reconnaissances Et Explorations,**3*, 675–699.

[CR5] Brown, J. (1995). Traditional metalworking in Kenya. *Cambridge monograph in African archaeology. * (Vol. 38). Oxford, Oxbow Books.

[CR6] Buchanan, F. (1807). *A journey from Madras through the countries of Mysore, Canara, and Malabar. *(Vol. 3). London.

[CR7] Célis, G. R. (1989). La métallurgie traditionnelle au Burundi, au Rwanda et au Buha : Essai de synthèse. *Anthropos,**83*(1.3), 25–46.

[CR8] Crew, P. (1991). The experimental production of prehistoric bar iron. *Historical Metallurgy,**5*, 21–36.

[CR9] de Barros, P. (2021). La Métallurgie du Fer en Pays Bassar (Nord-Togo) Depuis 2400 Ans*. *In* L’Âge du Fer Ancien (de 400 avant J.-C. à 130 après J.-C.) *(Vol. 1). Éditions Harmattans.

[CR10] de Barros, P., Iles, L., Frame, L. D., & Killick, D. (2020). The early iron metallurgy of Bassar, Togo: Furnaces, metallurgical remains and iron objects. *Azania: Archaeological Research in Africa,**55*(1), 3–43. 10.1080/0067270X.2020.1721841

[CR11] Deme, A. (2003). *Archaeological investigations of settlement and emerging complexity in the Middle Senegal Valley*. Unpublished Ph.D. Thesis, Rice University, Houston.

[CR12] Deme, A., & McIntosh, S. K. (2006). Excavations at Walalde: New light on the settlement of the Middle Senegal Valley by iron using peoples. *Journal of African Archaeology,**4*(2), 317–347.

[CR13] Dianifaba, L. (2020). *Produire et utiliser du fer au Gajaaga*. Thèse de doctorat, Université Paris Nanterre / Université Cheikh Anta Diop.

[CR14] Echard, N. (1983). Scories et Symboles : Remarques sur la métallurgie haussa du fer au Niger. In Echard, N. (Ed.) *Métallurgies Africaines. Nouvelles contributions *(Vol. 9, pp. 209–224). Mémoires de la société des Africanistes.

[CR15] Holl, A. F. C. (2009). West African early metallurgies: New evidence and old orthodoxy. *Journal of World Prehistory,**22*(4), 415–438.

[CR16] Holl, A. F. C. (2019). The dynamics of mounds-clusters in the Mouhoun Bend (Burkina Faso). *Journal of Anthropological and Archaeological Sciences,**1*(1), 1–12.

[CR17] Huysecom, É., Chevrier, B., Mayor, A., Pelmoine, T., Cantin, N., Chaix, L., Leplongeon, A., Lespez, L., Loukou, S., Rasse, M., Truffa Giachet, M., Bocoum, H., Camara, A., Hajdas, I., Lebrun, B., & Tribolo, C. (2016). Transitions et continuités dans la vallée de la Falémé (Sénégal) : résultats de la 18ème année de recherche du programme international « Peuplement humain et paléoenvironnement en Afrique ». In *SLSA Jahresbericht – Rapport annuel – Annual report 2015*. Zürich, Vaduz : Fondation Suisse-Liechtenstein pour les recherches archéologiques à l’étranger, p. 103–160.

[CR18] Huysecom, É., Jeanbourquin, C., Mayor, A., Chevrier, B., Loukou, S., Canetti, M., Diallo M., Bocoum, H., Gueye, N. S., Hajdas, I., Lespez, L., & Rasse, M. (2013). Reconnaissance dans la vallée de la Falémé (Sénégal oriental) : la 15ème année de recherches du programme international « Peuplement humain et paléoenvironnement en Afrique de l’Ouest ». In *SLSA Jahresbericht – Rapport annuel – Annual report 2012*. Zürich, Vaduz : Fondation Suisse-Liechtenstein pour les recherches archéologiques à l’étranger, p. 25–112.

[CR19] Huysecom, É., Loukou, S., Mayor, A., Jeanbourquin, C., Canetti, M., Chaix, L., Sanago, K., Garnier, A., Chevrier, B., Hajdas, I., Ballouche, A., Lespez, L., Pollarolo, L., Bocoum, H., Camara, A., Gueye, N. S., Gardiola-Figols, M., Sankaré, F., Timpoko-Kiénon-Kaboré, H., Rasse, M., Guindo, N., & Tribolo, C. (2015). La construction du cadre chronologique et paléoenvironnement de la moyenne vallée de la Falémé (Sénégal oriental) : les résultats de la 17ème année d’activité du programme international « Peuplement humain et paléoenvironnement en Afrique de l’Ouest *». In In SLSA Jahresbericht – Rapport annuel – Annual report 2014. *Zürich, Vaduz : Fondation Suisse-Liechtenstein pour les recherches archéologiques à l’étranger, p. 99–109.

[CR20] Huysecom, É., Loukou, S., Mayor, A., Jeanbourquin, C., Chaix, L., Chevrier, B., Ballouche, A., Bocoum, H., Gueye, N. S., Timpoko-Kiénon-Kaboré, H., Rasse, M., Tribolo, C. (2014). Vallée de la Falémé (Sénégal oriental) et Parc national des îles Eotilé (Côte d’Ivoire) : la 16ème année de recherche du programme « Peuplement humain et paléoenvironnement en Afrique ». In* SLSA Jahresbericht – Rapport annuel – Annual report 2013. *Zürich, Vaduz : Fondation Suisse-Liechtenstein pour les recherches archéologiques à l’étranger, p. 113–176.

[CR21] Iles, L. (2016). The role of metallurgy in transforming global forests. *Jourmal of Archaeological Method and Theory,**g*, 1219–1241. 10.1007/s10816-015-9266-7

[CR22] Junius, H. (2023). *Nok Eisen : zentralnigerianische Eisenverhüttung in der Mitte des ersten Jahrtausends vor Christus*. PhD Thesis, Universitätsbibliothek Johann Christian Senckenberg, Frankfurt am Main.

[CR23] Kiethega, J. B. (2006). *La métallurgie lourde du fer au Burkina Faso. Une technologie à l’époque précoloniale*. Karthala.

[CR24] Killick, D. (2004). Review essay: What do we know about African iron working? *Journal of African Archaeology,**2*(1), 97–112.

[CR25] Killick, D. (2016). A global perspective on the pyrotechnologies of Sub-Saharan Africa. *Azania : Archaeological Research in Africa,**51*(1), 62–87. 10.1080/0067270X.2016.1150082

[CR26] Killick, D., van der Merwe, J., Gordon, R. B., & Grébénart, D. (1988). Reassessment of the evidence for early metallurgy in Niger, West Africa. *Journal of Archaeological Science,**15*(4), 367–394. 10.1016/0305-4403(88)90036-2

[CR27] MacDonald, K. C., Vernet, R., Martinón-Torres, M., & Fuller, D. Q. (2009). Dhar néma: From early agriculture to metallurgy in southeastern Mauritania. *Azania : Archaeological Research in Africa,**44*(1), 3–48. 10.1080/00671990902811330

[CR28] Mayor, A., Douze, K., Lorenzo-Martinez,M., Truff a Giachet, M., Aymeric Nsangou, J., Bocoum, H., Champion, L., Cervera, C., Davidoux, S., Garnier, A., Hajdas, I., Lebrun, B., Lespez, L., Loukou, S., Mokadem, F., Ndiaye, M., Pelmoine, T., Rasse, M., Serneels, V., Tribolo, C., Virmoux, C., Walmsley, A., Huysecom, E. (2018). Dynamiques techniques et environnementales dans la vallée de la Falémé (Sénégal): Résultats de la 20ème année de recherche du programme «Peuplement humain et paléoenvironnement en Afrique». In *SLSA Jahresbericht – Rapport annuel – Annual report* 2017. Zürich, Vaduz : Fondation Suisse-Liechtenstein pour les recherches archéologiques à l’étranger, p. 157–256.

[CR29] Mayor, A., Douze, K., Aymeric, J., Bocoum, H., Cantin, N., Cervera, C., Champion, L., Chiquet, P., Davidoux, S.,Garnier, A., Hajdas, I., Kante, C.O., Lebrun, B., Lespez, L., NDiaye, M., Ollier, C., Pelmoine, T., Rasse, M., Tribolo, C.,Huysecom, E. (2019). Archéologie et environnements au Sénégal oriental. Résultats de la 21ème année duprogramme international « Peuplement humain et paléoenvironnement en Afrique ». In *SLSA Jahresbericht – Rapport annuel – Annual report* 2018. Zürich, Vaduz : Fondation Suisse-Liechtenstein pour les recherches archéologiques àl’étranger, p. 185–266.

[CR30] Mayor, A., K. Douze, H. Bocoum, C. Cervera, L. Champion, D. Glauser, N. Guindo, G. Kassogué, M. Ndiaye, M. Rasse,B. Traoré, É. Huysecom. (2020) « Archéologie dans la Falémé (Sénégal oriental): résultats de la 22ème année duprogramme ‹Peuplement humain et paléoenvironnement enAfrique. » In *SLSA Jahresbericht – Rapport annuel –Annual report* 2019. Zürich, Vaduz : Fondation Suisse-Liechtenstein pour les recherches archéologiques à l’étranger, p. 197–224.

[CR31] Mayor, A., Douze, K., Bocoum, H., Ververa, C., Champion, L., Chiquet, P., Dianifaba, L., Hajdas, I., Koffi, S., Lamotte, P., Lespez, L., NDiaye, M., Rasse, M., Schmid, V., Touré, A., Tribolo, C., Walmsley, A., & Huysecom, E. (2021). Évolutions techniques sur la longue durée dans la Falémé. Résultats de la 23ème année du programme « Peuplement humain et paléoenvironnement en Afrique ». In *SLSA Jahresbericht – Rapport annuel – Annual report* 2020. Zürich, Vaduz : Fondation Suisse-Liechtenstein pour les recherches archéologiques à l’étranger, p. 149–218.

[CR32] Mayor, A., Douze, K., Pruvost, C., Bocoum, H., Cervera, C., Champion, L., Chiquet, P., Hajdas, I., Kehl, V., Lamotte, P., Lespez, L., NDiaye, M., Rasse, M., Serneels, V., Tribolo, C., Huysecom, E. (2022). Dynamiques techniques de l’Acheuléen à la période des Grands Empires dans la vallée de la Falémé, Sénégal. Résultats de la 24ème année du programme « Peuplement humain et paléoenvironnement en Afrique ». In *SLSA Jahresbericht – Rapport annuel – Annual report* 2021. Zürich, Vaduz : Fondation Suisse-Liechtenstein pour les recherches archéologiques à l’étranger, p. 77–149.

[CR33] Mayor, A., Douze, K., Pruvost, C., Bocoum, H., Cervera, C., Hajdas, I., Kehl, V., Lamotte, P., Ndiaye, M., Huysecom, E. (2023). De la pierre au fer dans la vallée de la Falémé, Sénégal : résultats de la 25ème année du programme « Peuplement humain et paléoenvironnement en Afrique ». In *SLSA Jahresbericht – Rapport annuel – Annual report 2022*. Fondation Suisse-Liechtenstein pour les recherches archéologiques à l’étranger, p. 73-106.

[CR34] Mayor, A., Pruvost, C., Ben Arous, E., Bocoum, H., Diédhiou, I., Dione, S., Hajdas, I., Höhn, A., Katona-Serneels, I., Morel, M., Ndiaye, M., Ndoye, K., Serneels, V., & Tribolo, C. (2025). Valorisation des découvertes archéologiques de la Falémé (Sénégal) : résultats de la 27ème année du programme « Peuplement humain et paléoenvironnement en Afrique ». In *SLSA Jahresbericht – Rapport annuel – Annual report 2024. *Zürich, Vaduz : Fondation Suisse-Liechtenstein pour les recherches archéologiques à l’étranger, p. 181-227.

[CR35] Montes-Landa, J., Timberlake, S., & Martinón-Torres, M. (2024). Debunking deterministic narratives of technological development through experimentation: A critical review of the prehistory of tin bronze alloying. *Journal of Archaeological Method and Theory,**31*, 2012–2052. 10.1007/s10816-024-09661-w

[CR36] Morel, M. (2023). *Iron metallurgy in northeastern Madagascar: A study of Rasikajy metallurgical production between the 11th and 15th**century*, Université de Fribourg, Dep. Geosciences, Librum, Basel — Frankfurt a. M.

[CR37] Morel, M., Lamotte, P., Serneels, V., & Mayor, A. (In preparation). A Thousand-Year Iron Smelting Tradition in the Falémé Valley: Technical Evolution and Chemical Variability of Slag.

[CR38] Morel, M., & Serneels, V. (2021). Interpreting the chemical variability of iron smelting slag: A case study from Northeastern Madagascar. *Minerals,**11*, 900.

[CR39] Morel, M., Serneels, V., Robion-Brunner, C., & Timpoko Kiénon Kabore, H. (2025). Efficiency of ancient iron production: A case study from the Kaniasso district, Ivory Coast (11th-19th century). *Journal of Archaeological Science: Reports,**63*, 105089. 10.1016/j.jasrep.2025.105089

[CR40] Okafor, E. E. (2002). La réduction du fer dans les Bas-Fourneaux—Une industrie vieille de 2500 ans au Nigeria. In H. Bocoum (Ed.), *Aux origines de la métallurgie du fer en Afrique: : une ancienneté méconnue, Afrique de l'Ouest et Afrique centrale*. Editions UNESCO, Paris, p. 35-48.

[CR41] Okafor, E. E., & Phillips, P. (1992). New 14C ages from Nsukka, Nigeria, and the origins of African metallurgy. *Antiquity,**66*(252), 686–688. 10.1017/S0003598X00039387

[CR42] Ollier, C. (2018). *Pour un usage des photographies aériennes à basse altitude*. Exemple de la méthode appliquée dans le cadre de la mission PHPA 2018. Master 1 (Ecole normale supérieure de Lyon), Université Lumière Lyon 2 (unpublished).

[CR43] Pelet, P.-L. (1974). Une industrie bimillénaire : La sidérurgie du Jura vaudois. *Annales. Histoire, Sciences Sociales,**29*(4), 789–812. 10.3406/ahess.1974.293515

[CR44] Perret, S., Serneels, V. (2009). Technological characterisation and quantification of a large-scale iron smelting site in Fiko (dogon plateau, Mali). In *Proceedings of the 36th International Symposium on Archaeometry,* Quebec City (May 2006). 453–463.

[CR45] Quéchon, G., & Roset, J. P. (1974). Prospection archéologique du Massif de Termit, Niger. *Cahiers ORSTOM. Série Sciences Humaines,**11*, 85–104.

[CR46] Robert-Chaleix, D., & Sognane, M. (1983). Une industrie métallurgique ancienne sur la rive mauritanienne du fleuve Sénégal. In Textes réunis par Nicole Echard *Métallurgies africaines : nouvelles contributions.* Paris, Société des Africanistes, p. 45–62.

[CR47] Robion-Brunner, C. (2018*). L’Afrique des métaux.* In F.-X. Fauvelle (Ed.*), L’Afrique ancienne. De l’Acacus au Zimbabwe, 20000 avant notre ère - XVIIe siècle*, Paris, Belin (Mondes anciens), p. 516‑543, 978-2-7011-9836-1.

[CR48] Robion-Brunner, C., Perret, S., Serneels, V., Dembélé, A., Huysecom, E. (2006). A thousand years of iron metallurgy on the Dogon plateau (Mali). In *Proceedings of the 18th Biennial Meeting of the Society of Africanist Archaeologists*. Calgary (Canada).

[CR49] Robion-Brunner, C., & Serneels, V. (2017). Protéger et fouiller un site archéologique : les sites métallurgiques. In Livingstone Smith, A., Cornelissen, E., Gosselain, O. P., MacEachern, S. (Eds.), *Manuel de terrain en archéologie africaine*, Tervuren, Musée royal de l’Afrique centrale (« Documents de sciences humaines et sociales »), p. 129–133, 978-9-4922-4428-4.

[CR50] Serneels, V., Donadini, F., Kiénon-Timpoko, H., Koté, L., Kouassi, S. K., Ramseyer, D., & Simpore, L. (2014). Origine et développement de la métallurgie du fer au Burkina Faso et en Côte d’Ivoire. Avancement des recherches en 2013 et quantification des vestiges de Korsimoro (Burkina Faso). *Jahresbericht SLSA,**2013*, 65–112.

[CR51] Serneels, V., Timpoko Kiénon-Kaboré, H., Koté, L., Kouassi, S. K., Mauvilly, M., Ramseyer, D., & Simporé, L. (2013). Origine et développement de la métallurgie du fer au Burkina Faso et en Côte d’Ivoire. Premiers résultats sur le site sidérurgique de Siola (Kaniasso, Denguélé, Côte d’Ivoire). *SLSA Jahresbericht,**2012*, 113–143.

[CR52] Serneels, V., Timpoko Kiénon-Kaboré, H., Koté, L., Kouassi, S. K., Ramseyer, D., & Simporé, L. (2012). Origine et développement de la métallurgie du fer au Burkina Faso et en Côte d’Ivoire. Premiers résultats sur le site sidérurgique de Korsimoro (Sanmatenga, Burkina Faso). *Jahresbericht SLSA,**2011*, 2–32.

[CR53] Thiaw, I. (1999). *An archaeological investigation of long-term culture change in the Lower Falemme (Upper Senegal Region), A.D. 500–1900*. PhD Thesis, Rice University, Department of Anthropology, Houston, TX, United States.

[CR54] Walmsley, A. (2018). *Archéométallurgie du fer dans la Réserve du Boundou (Sénégal oriental). Nouvelles données pour un âge du fer ancien en Afrique de l’ouest*. Mémoire de master en archéologie préhistorique, Faculté des sciences, Université de Genève.

[CR55] Walmsley, A., Serneels, V., Hajdas, I., & Mayor, A. (2020). Variability of early iron production in the Falémé Valley Region, Eastern Senegal. *African Archaeological RevIew,**37*, 225–250. 10.1007/s10437-020-09381-y

